# Estimating neutrosophic finite median employing robust measures of the auxiliary variable

**DOI:** 10.1038/s41598-024-60714-2

**Published:** 2024-05-04

**Authors:** Saadia Masood, Bareera Ibrar, Javid Shabbir, Ali Shokri, Zabihullah Movaheedi

**Affiliations:** 1 Department of Statistics, PMAS-University of Arid Agriculture, Rawalpindi, Pakistan; 2 Department of Statistics, University of Wah, Wah, Pakistan; 3 Department of Mathematics, Faculty of Science, University of Maragheh, Maragheh, 83111-55181 Iran; 4 Department of Mathematics, Faculty of Science, Herat University, Herat, 3001 Afghanistan

**Keywords:** Neutrosophic statistics, Auxiliary variable, Population median, Robust measures, Mean squared error, Applied mathematics, Computational science, Information technology, Scientific data, Statistics

## Abstract

Our study explores neutrosophic statistics, an extension of classical and fuzzy statistics, to address the challenges of data uncertainty. By leveraging accurate measurements of an auxiliary variable, we can derive precise estimates for the unknown population median. The estimators introduced in this research are particularly useful for analysing unclear, vague data or within the neutrosophic realm. Unlike traditional methods that yield single-valued outcomes, our estimators produce ranges, suggesting where the population parameter is likely to be. We present the suggested generalised estimator's bias and mean square error within a first-order approximation framework. The practicality and efficiency of these proposed neutrosophic estimators are demonstrated through real-world data applications and the simulated data set.

## Introduction

Significant strides have been made in recent years in estimating the finite population mean, proportion, and variance using auxiliary information. However, the median, a more robust measure than the mean in the face of exceptionally low or high values, has received attention. Our research stands out by proposing a practical solution for estimating the median of a study variable for a finite population, even in the presence of neutrosophic forms of research and supporting variables with extreme values or outliers.

Several studies have made significant contributions to estimating a finite population's median. Gross^1^ used the sample median estimator in various sampling methods. Kuk and Mak^2^ proposed using the known median of the auxiliary variable to estimate the median of the study variable. Francisco and Fuller^3^ approximated the distribution function of a finite population using the median. Smarandache^4^ advocated using Neutrosophic statistics in uncertain systems. Singh et al.^5^ suggested a generalized family of median estimators in double sampling. These studies have laid the foundation for our research, which aims to build upon these findings and propose a practical solution for estimating the median of a study variable for a finite population.

A few chain ratio-type estimators were introduced by^6^ using the additional knowledge of the range of the auxiliary variable, whereas^7^ discussed the transformed ratio-type estimator using 's^8^ idea. Shokri^9^, ^10^, ^58^ presented new approaches to solving second-order initial value problems, providing effectiveness in addressing computational challenges. A generalized median estimator utilizing the transformed auxiliary variable was addressed by ^11^. Lamichhane et al.^12^ suggested a unique estimation for the finite population mean using the auxiliary variable's median.

Smarandache^13^ suggested that the sample size may not be accurately specified in neutrosophic statistics and hence presented the neutrosophic logic may fall within the interval $[a, \, b]$ (unidentified exactly). Sahin^14^ and Şahin^15^ proposed a new similarity measure based on falsity value between single-valued neutrosophic sets based on the centroid points of transformed single-valued neutrosophic numbers in decision-making. Shokri^16^ and Uluçay^17^ proposed the similarity measures of bipolar neutrosophic sets and their application to multiple criteria decision-making. Jan^18^ proposed multi-criteria decision-making for cubic linguistic information. Aslam^19^,^20^ explained the Neutrosophic analysis of variance on neutrosophic data. Using two supplementary variables,^21^ and^22^ presented difference-type median estimators for obtaining the population median. Chakraborty^23^ developed and categorized a trapezoidal bipolar neutrosophic number in decision-making.

For instance,^24^'s development of complex neutrosophic fuzzy sets contributed to advancing the field of fuzzy sets. In addition, they offered an extensive flowchart of fuzzy sets with extensions, a description of their properties, and an explanation of how interval-valued neutrosophic sets function. Haque^25^ proposed a multi-criteria group decision-making strategy for the cylindrical neutrosophic domain.

Data visualization, analysis, and inference have long used classical statistics (CS). The CS investigates the assumption of data certainty. When the observations' measurements are precise, new methods for handling uncertain data are needed. Fuzzy logic works with data where the variable being researched lacks accurate measurements. They are quickly evolving and often used in settings where decisions are made. Fuzzy statistics examine data with ambiguous, opaque, or uncertain observations but neglect indeterminacy measurement. In this situation, showing a range of specific observations might be possible. The data included inside the indeterminacy interval cannot thus be analyzed using the CS. Neutrosophic statistical techniques are used to analyze the ambiguous neutrosophic data. The neutrosophic logic is used to interpret vague or unclear observations and allows for the measurement of indeterminacy and the determinate part of the observations.

Aslam^26^,^27^ elaborated on Neutrosophic Interval Statistics (NIS), Neutrosophic Applied Statistics (NAS), and Neutrosophic Statistical Quality control (NSQC), respectively. Uluçay^28^ suggested the idea of interval-valued refined neutrosophic sets and their applications. A large number of neutrosophic sets are described in the literature.

Tahir^29^ addressed a sampling gap by conducting a study to estimate a population's characteristics in a neutrosophic environment. They presented estimators of the neutrosophic ratio-type for estimating the finite population mean utilizing the additional information. Uluçay^30^,^28^ presented Q-neutrosophic soft graphs in operations management and communication networks. Vishwakarma and Singh^31^ proposed a neutrosophic ranked set sampling strategy for estimating the population mean under uncertainty using neutrosophic auxiliary information.

Table 1 illustrates the versatility and utility of neutrosophic logic in tackling problems across various disciplines, especially in situations where traditional binary logic falls short due to vagueness, uncertainty, or contradictory information.

**Table 1:** Some real-life problems and domains under neutrosophic logic.

Domain	Problem solved	Description
Medical diagnosis	Disease identification	Improving the accuracy of diagnosing diseases by handling uncertain or incomplete medical data
Image processing	Image segmentation and enhancement	Enhancing image quality and segmentation in environments with vague and indistinct boundaries
Decision making	Multi-criteria decision making (MCDM)	Facilitating decision-making processes in scenarios with incomplete, inconsistent, or uncertain information
Engineering	Fault diagnosis and system reliability analysis	Assessing system reliability and diagnosing faults in complex engineering systems under uncertainty
Environmental science	Environmental impact assessment	Evaluating environmental impacts with imprecise data, aiding in more effective decision-making
Social sciences	Opinion mining and sentiment analysis	Analyzing sentiments and opinions in social media data, where opinions can be indeterminate or inconsistent
Robotics	Robot localization and navigation	Improving robot navigation in uncertain environments by dealing with imprecise sensor data
Economics	Economic forecasting	Enhancing economic forecasting models by incorporating uncertainty in economic data

Despite an extensive review of existing research, a study still needs to address the challenge of estimating the unknown population median in survey sampling when additional variables are introduced under neutrosophic information. The effectiveness of estimators remains to be determined in scenarios where the study variable and supporting variables take on neutrosophic forms, and the dataset includes extreme values or outliers. The lack of a practical solution for median estimation in such cases underscores the novelty and importance of our proposed neutrosophic median estimation method based on reliable measures of the auxiliary variable that are already known.

Neutrosophic statistics are applied in decision-making, risk assessment, uncertainty modelling, image processing, medical diagnosis, finance, and engineering for robust analysis as discussed in Table 1.

The paper is organized in a way that Section "The neutrosophic statistics" elaborates the details of Neutrosophic statistics along with symbols and notations. The adapted and proposed Neutrosophic median estimators along with the efficiency comparison are given in Section "The neutrosophic median estimators under simple random sampling". The numerical and graphical results related to real-life and simulated data sets are presented in Section "Real-life application". Finally, the proposed work is concluded in Section "Conclusion".

## The neutrosophic statistics

Neutrosophic statistics, a unique approach, are designed to handle datasets with a degree of ambiguity or partial information. This method allows for conflicting beliefs and accommodates a range of uncertain numbers that may represent some observations, including an exact measurement. In contrast, traditional statistics falter when faced with uncertainty. This is where the intriguing potential of neutrosophic statistics comes into play, offering a fresh perspective on data analysis.

In real-world problems, the population parameters are often unknown. In such cases, statistical inference methods may need to be more practical. Instead, acceptable estimates are used, resolving the issue of an unknown parameter value by estimating its values. This pragmatic approach reassures the statistician that the derived data are vague but still useful. Neutrosophic statistics, with their ability to calculate the best interval value with the minimum mean square error, offer a reliable solution to these problems.

Previous study efforts provided a limited range of neutrosophic observations, including quantifiable neutrosophic data. Furthermore^32–40^ discussed numerous approaches, such as interval-based approaches, Triangular or trapezoidal fuzzy numbers, and single-valued fuzzy numbers, exist to express the range of neutrosophic numbers along with Optimal trajectories in reproduction models of economic dynamics. He^41^ proposed a fractal model for internal temperature response in porous concrete, advancing understanding in applied mathematics. Iskandarov and Komartsova^42,57^ investigated integral perturbations' influence on boundedness in fourth-order linear differential equations. Khankishiyev^43^ employed finite differences to solve loaded differential equations, while ^44^, ^56^ explored dark energy solutions without a cosmological constant. Furthermore,^45^ established conditions for complete monotonicity in the differential functions involving trigamma functions.

Let the neutrosophic range is $T_{N} = T_{L} + T_{U} \ell_{N}$ with $\ell_{N} \in [\ell_{L} , \, \ell_{U} ]$, the neutrosophic variable $T_{N}$ indicates the neutrosophic samples selected from a population having imprecise, ambiguous and unclear measurements. Thus, for the neutrosophic data in the interval form, we use notation $T_{N} \in [a, \, b],$ where $a$ and $b$ are the lower and upper values of the neutrosophic data, respectively.

**Figure 1:**
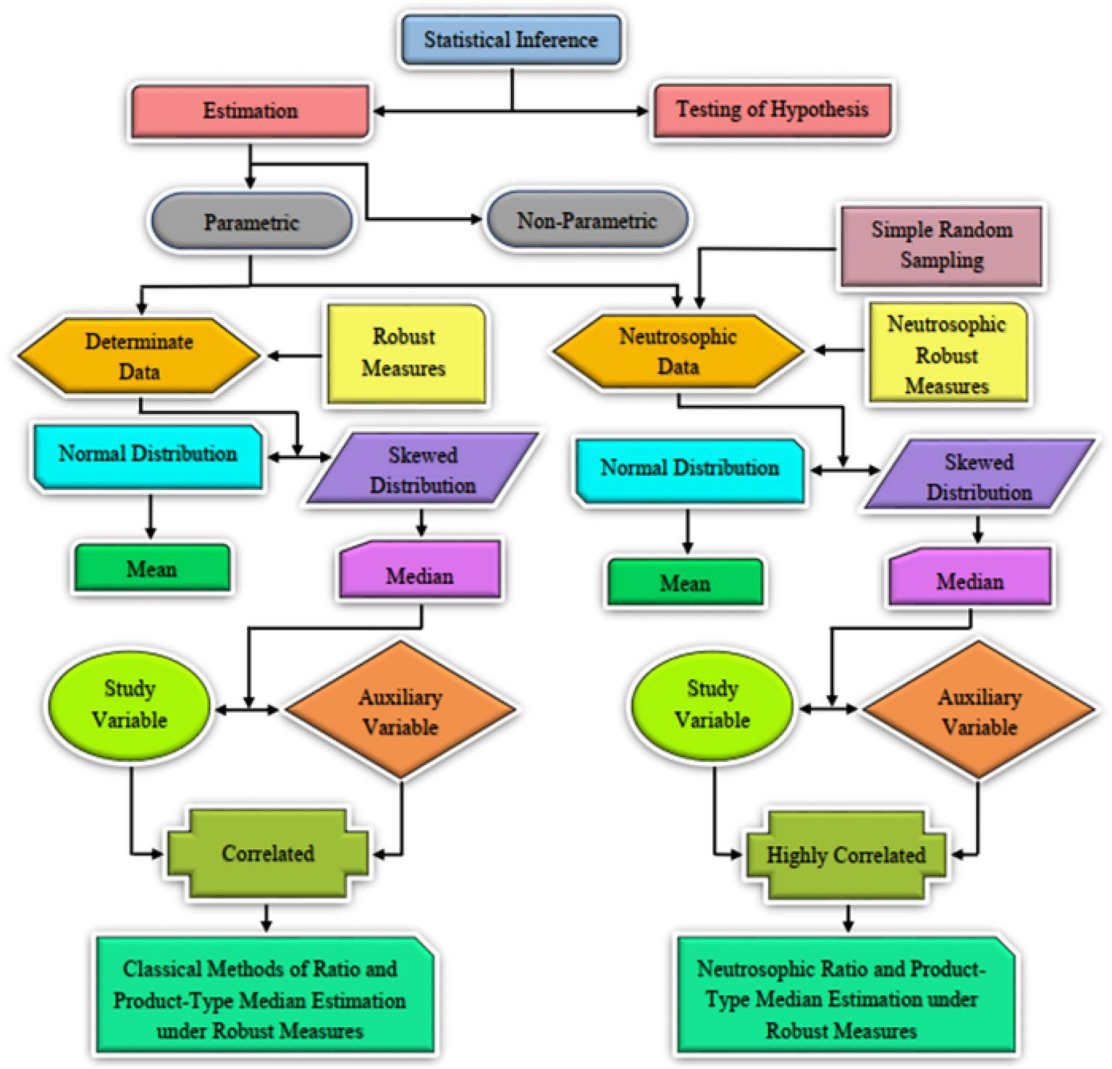
Workflow of the parameter estimation.

Figure 1 depicts the approach to applying the proposed estimation methods in neutrosophic statistics. This workflow developed a few neutrosophic estimators to estimate the finite population median in the presence of supplementary data, which are well suited for overcoming the sample indeterminacy problem.

### Symbols and notations

Suppose a neutrosophic random sample of size $$n_{N} \in [n_{L} , \, n_{U} ],$$ selected from a finite population $$U = \left\{ {U_{1} ,U_{2} ,...,U_{N} } \right\}$$ of $$N$$ recognisable units. Assume $$y_{N} (i)$$ and $$x_{N} (i),$$$$i = (1,2,...,n)$$ represent the $$i^{th}$$ sampled unit's values of the neutrosophic data of the study variable $$y_{N(i)} \in [y_{L} , \, y_{U} ]$$ and the supplementary variable $$x_{N(i)} \in [x_{L} , \, x_{U} ]$$, respectively. Let $$M_{yN}$$ be the neutrosophic variable of interest and $$\hat{M}_{yN}$$ and $$\hat{M}_{xN}$$ represent the sample median that corresponds to the population median $$M_{yN}$$ and $$M_{xN}$$ respectively with the probability density functions $$f_{yN} (M_{yN} )$$ and $$f_{xN} (M_{xN} ),$$ respectively, where $$\hat{M}_{yN} \in [\hat{M}_{yL} , \, \hat{M}_{yU} ]$$ and $$\hat{M}_{xN} \in [\hat{M}_{xL} , \, \hat{M}_{xU} ].$$ Let $$\rho_{yxN} = \rho_{{(\hat{M}_{yN} ,\hat{M}_{xN} )}} = 4\rho_{11} (y_{N} ,x_{N} ) - 1$$ represent the neutrosophic population correlation coefficient between the neutrosophic sample medians ranging from $$- 1$$ to $$+ 1$$ as $$\rho_{11}$$ increases from 0 to 0.5, where $$\rho_{yxN} \in [\rho_{yxL} , \, \rho_{yxU} ]$$ such that $$P_{11} (y_{N} ,x_{N} ) = P(y_{N} \le M_{yN} \cap x_{N} \le M_{xN} ).$$ Similarly, $$Bias(\hat{M}_{yN} ) \in [Bias_{L} , \, Bias_{U} ]$$ and $$MSE(\hat{M}_{yN} ) \in [MSE_{L} , \, MSE_{U} ]$$ belong to the neutrosophic sets.

To get the characteristics of estimators, the relative error terms are defined as follows: Suppose $e_{{0N}} = (\hat{M}_{{yN}} - M_{{yN}} )/M_{{yN}}$ $e_{1N} = (\hat{M}_{xN} - M_{xN} )/M_{xN}$ are the neutrosophic errors where $e_{0N} \in [e_{0L} , \, e_{0U} ]$ and $e_{1N} \in [e_{1L} , \, e_{1U} ]$ such that $E(e_{iN} ) = 0 \, (i = 0,1),$ $E(e_{0N}^{2} ) = \lambda_{N} C_{{M_{yN} }}^{2} ,$ $E(e_{1N}^{2} ) = \lambda_{N} C_{{M_{xN} }}^{2} ,$ $E(e_{0N} e_{1N} ) = \lambda_{N} C_{{M_{yxN} }} ,$ where $C_{{M_{yxN} }} = \rho_{yxN} C_{{M_{yN} }} C_{{M_{xN} }} ,$ $C_{{M_{yN} }} = \left\{ {M_{yN} f_{yN} (M_{yN} )} \right\}^{ - 1} ,$ $C_{{M_{xN} }} = \left\{ {M_{xN} f_{xN} (M_{xN} )} \right\}^{ - 1}$ are the neutrosophic coefficients of variation, respectively. Let $C_{{M_{yxN} }} \in [C_{{M_{yxL} }} , \, C_{{M_{yxU} }} ]$, $C_{{M_{yN} }} \in [C_{{M_{yL} }} , \, C_{{M_{yU} }} ]$,$C_{{M_{xN} }} \in [C_{{M_{xL} }} , \, C_{{M_{xU} }} ]$ and $\lambda_{N} = \frac{1}{4}\left( {\frac{1}{{n_{N} }} - \frac{1}{{N_{N} }}} \right),$ where $\lambda_{N} \in [\lambda_{L} , \, \lambda_{U} ]$.

## The neutrosophic median estimators under simple random sampling

First, we present a few adapted neutrosophic median estimators using auxiliary information under simple random sampling to address uncertainty and neutrosophic data.

### Adapted median estimators with auxiliary variable

(i) Motivated by^1^, we propose a neutrosophic traditional median estimator and its variance, along with the expression of variance is given by
  1 $$\hat{M}_{0N} = \hat{M}_{yN}$$
  2 $$Var(\hat{M}_{0N} ) = \lambda_{N} M_{yN}^{2} C_{{M_{yN} }}^{2}$$
(ii) Motivated by^2^, we developed a novel neutrosophic traditional ratio estimator, along with the expressions of Bias and MSE are
  3 $$\hat{M}_{RN} = \hat{M}_{yN} \left( {\frac{{M_{xN} }}{{\hat{M}_{xN} }}} \right)$$
  4 $$Bias(\hat{M}_{RN} ) \cong \lambda_{N} M_{yN} \left\{ {C_{{M_{xN} }}^{2} - C_{{M_{yxN} }} } \right\}$$

and

5 $$MSE(\hat{M}_{RN} ) \cong \lambda_{N} M_{yN}^{2} \left\{ {C_{{M_{yN} }}^{2} + C_{{M_{xN} }}^{2} - 2C_{{M_{yxN} }} } \right\}$$

The ratio estimator ($\hat{M}_{RN}$) performs better than $\hat{M}_{0N}$ if $\rho_{yxN} > 0.5\frac{{C_{{M_{xN} }} }}{{C_{{M_{yN} }} }}.$

(iii) Motivated by^46^, the neutrosophic exponential ratio-type estimator, along with the expressions of Bias and MSE are given by
  6 $$\hat{M}_{EN} = \hat{M}_{yN} \exp \left( {\frac{{M_{xN} - \hat{M}_{xN} }}{{M_{xN} + \hat{M}_{xN} }}} \right)$$
  7 $$Bias(\hat{M}_{EN} ) \cong M_{yN} \lambda_{N} \left( {\frac{3}{8}C_{{M_{xN} }}^{2} - \frac{1}{2}C_{{M_{yxN} }} } \right)$$

and

8 $$MSE(\hat{M}_{EN} ) \cong M_{yN}^{2} \lambda_{N} \left( {C_{{M_{yN} }}^{2} + \frac{1}{4}C_{{M_{xN} }}^{2} - C_{{M_{yxN} }} } \right)$$

The exponential ratio estimator ($\hat{M}_{EN}$) is more efficient than $\hat{M}_{0N}$ and $\hat{M}_{RN}$ if $\rho_{yxN} > 0.25\frac{{C_{{M_{xN} }} }}{{C_{{M_{yN} }} }}$ and $\rho_{yxN} < 0.75\frac{{C_{{M_{xN} }} }}{{C_{{M_{yN} }} }},$ respectively.

(iv) The adapted neutrosophic difference estimator along with the expression of variance is given by
  9 $$\hat{M}_{{D_{0N} }} = \hat{M}_{yN} + d_{0N} (M_{xN} - \hat{M}_{xN} )$$

At the optimal value of $d_{0N} ,$ which is $d_{0N(opt)} = \frac{{M_{yN} \rho_{yxN} C_{{M_{yN} }} }}{{M_{xN} C_{{M_{xN} }} }},$ the minimum MSE of $\hat{M}_{{D_{0N} }} ,$ is given by

10 $$Var(\hat{M}_{{D_{0N} }} )_{\min } \cong M_{yN}^{2} C_{{M_{yN} }}^{2} \lambda_{N} \left( {1 - \rho_{yxN}^{2} } \right)$$

(xxii) Adapted from^22^, difference-type estimators, along with the expressions of Bias and minimum mean square errors, are given by
  11 $$\hat{M}_{{D_{1N} }} = d_{1N} \hat{M}_{yN} + d_{2N} (M_{xN} - \hat{M}_{xN} )$$
  12 $$\hat{M}_{{D_{2N} }} = \left\{ {d_{3N} \hat{M}_{yN} + d_{4N} (M_{xN} - \hat{M}_{xN} )} \right\}\left( {\frac{{M_{xN} }}{{\hat{M}_{xN} }}} \right),$$
  13 $$\hat{M}_{{D_{3N} }} = \left\{ {d_{5N} \hat{M}_{yN} + d_{6N} (M_{xN} - \hat{M}_{xN} )} \right\}\exp \left( {\frac{{M_{xN} - \hat{M}_{xN} }}{{M_{xN} + \hat{M}_{xN} }}} \right),$$

and

14 $$\hat{M}_{{D_{4N} }} = \left\{ {d_{7N} \hat{M}_{yN} + d_{8N} (M_{xN} - \hat{M}_{xN} )} \right\}\exp \left( {\frac{{M_{xN} }}{{\hat{M}_{xN} }} - 1} \right),$$

where $d_{iN} (i = 1 - 8)$ are constants determined below by optimality considerations as

$$d_{1N(opt)} = \frac{{B_{0N} }}{{A_{0N} B_{0N} - C_{0N}^{2} + B_{0N} }},$$ $$d_{2N(opt)} = \frac{{M_{yN} }}{{M_{xN} }}\frac{{C_{0N} }}{{A_{0N} B_{0N} - C_{0N}^{2} + B_{0N} }},$$

$$d_{3N(opt)} = \frac{{B_{1N} (C_{1N} - D_{1N} + 1)}}{{A_{1N} B_{1N} - D_{1N}^{2} + B_{1N} }},$$ $$d_{4N(opt)} = \frac{{M_{yN} }}{{M_{xN} }}\frac{{(A_{1N} B_{1N} - C_{1N} D_{1N} + B_{1N} - D_{1N} )}}{{(A_{1N} B_{1N} - D_{1N}^{2} + B_{1N} )}},$$

$$d_{5N(opt)} = \frac{{(B_{2N} C_{2N} - D_{2N} E_{2N} + B_{2N} )}}{{(A_{2N} B_{2N} - E_{2N}^{2} + B_{2N} )}},$$ $$d_{6N(opt)} = \frac{{M_{yN} }}{{M_{xN} }}\frac{{(A_{2N} D_{2N} - C_{2N} E_{2N} + D_{2N} - E_{2N} )}}{{(A_{2N} B_{2N} - E_{2N}^{2} + B_{2N} )}},$$

$$d_{7N(opt)} = \frac{{B_{3N} (C_{3N} - D_{3N} + 1)}}{{A_{3N} B_{3N} - D_{3N}^{2} + B_{3N} }},$$ $$d_{8N(opt)} = \frac{{M_{yN} }}{{M_{xN} }}\frac{{(A_{3N} B_{3N} - C_{3N} D_{3N} + B_{3N} - D_{3N} )}}{{(A_{3N} B_{3N} - D_{3N}^{2} + B_{3N} )}},$$where $$A_{0N} = \lambda_{N} C_{{M_{yN} }}^{2} ,$$ $$B_{0N} = \lambda_{N} C_{{M_{xN} }}^{2} ,$$ $$C_{0N} = \lambda_{N} C_{{M_{yxN} }} ,$$ $$A_{1N} = \lambda_{N} (C_{{M_{yN} }}^{2} + 3C_{{M_{xN} }}^{2} - 4C_{{M_{yxN} }} ),$$ $$B_{1N} = \lambda_{N} C_{{M_{xN} }}^{2} ,$$ $$C_{1N} = \lambda_{N} (C_{{M_{xN} }}^{2} - C_{{M_{yxN} }} ),$$ $$D_{1N} = \lambda_{N} (2C_{{M_{xN} }}^{2} - C_{{M_{yxN} }} ),$$ $$A_{2N} = \lambda_{N} (C_{{M_{yN} }}^{2} + C_{{M_{xN} }}^{2} - 2C_{{M_{yxN} }} ),$$ $$B_{2N} = \lambda_{N} C_{{M_{xN} }}^{2} ,$$ $$C_{2N} = \lambda_{N} \left( {\frac{3}{8}C_{{M_{xN} }}^{2} - \frac{1}{2}C_{{M_{yxN} }} } \right),$$ $$D_{2N} = \lambda_{N} C_{{M_{xN} }}^{2} /2,$$ $$E_{2N} = \lambda_{N} (C_{{M_{xN} }}^{2} - C_{{M_{yxN} }} ),$$ $$A_{3N} = \lambda_{N} (C_{{M_{yN} }}^{2} + 4C_{{M_{xN} }}^{2} - 4C_{{M_{yxN} }} ),$$$$B_{3N} = \lambda_{N} C_{{M_{xN} }}^{2} ,$$ $$C_{3N} = \lambda_{N} \left( {\frac{3}{2}C_{{M_{xN} }}^{2} - C_{{M_{yxN} }} } \right)$$ and $$D_{3N} = \lambda_{N} (2C_{{M_{xN} }}^{2} - C_{{M_{yxN} }} ).$$

15 $$Bias(\hat{M}_{{D_{1N} }} ) \cong (d_{1N} - 1)M_{yN} ,$$

16 $$Bias(\hat{M}_{{D_{2N} }} ) \cong (d_{3N} - 1)M_{yN} + d_{3N} M_{yN} C_{1N} + d_{4N} M_{xN} B_{1N} ,$$

17 $$Bias(\hat{M}_{{D_{3N} }} ) \cong (d_{5N} - 1)M_{yN} + d_{5N} M_{yN} C_{2N} + d_{6N} M_{xN} D_{2N} ,$$

18 $$Bias(\hat{M}_{{D_{4N} }} ) \cong (d_{7N} - 1)M_{yN} + d_{7N} M_{yN} C_{3N} + d_{8N} M_{xN} B_{3N} ,$$

19 $$MSE(\hat{M}_{{D_{1N} }} )_{\min } \cong M_{yN}^{2} \left\{ {1 - \frac{{B_{0N} }}{{A_{0N} B_{0N} - C_{0N}^{2} + B_{0N} }}} \right\},$$

20 $$MSE(\hat{M}_{{D_{2N} }} )_{\min } \cong M_{yN}^{2} \left\{ {1 - \frac{{A_{1N} B_{1N}^{2} + B_{1N} C_{1N}^{2} - 2B_{1N} C_{1N} D_{1N} + B_{1N}^{2} + 2B_{1N} C_{1N} - 2B_{1N} D_{1N} + B_{1N} }}{{A_{1N} B_{1N} - D_{1N}^{2} + B_{1N} }}} \right\},$$

21 $$MSE(\hat{M}_{{D_{3N} }} )_{\min } \cong M_{yN}^{2} \left\{ {1 - \frac{{A_{2N} D_{2N}^{2} + B_{2N} C_{2N}^{2} - 2C_{2N} D_{2N} E_{2N} + 2B_{2N} C_{2N} + D_{2N}^{2} - 2D_{2N} E_{2N} + B_{2N} }}{{A_{2N} B_{2N} - E_{2N}^{2} + B_{2N} }}} \right\},$$

and

22 $$MSE(\hat{M}_{{D_{4N} }} )_{\min } \cong M_{yN}^{2} \left\{ {1 - \frac{{A_{3N} B_{3N}^{2} + B_{3N} C_{3N}^{2} - 2B_{3N} C_{3N} D_{3N} + B_{3N}^{2} + 2B_{3N} C_{3N} - 2B_{3N} D_{3N} + B_{3N} }}{{A_{3N} B_{3N} - D_{3N}^{2} + B_{3N} }}} \right\}.$$

### The proposed generalized neutrosophic median estimator

Traditional estimators, often hindered by their reliance on historical data, struggle with accuracy, particularly with outliers. This section introduces advanced neutrosophic estimators for accurately predicting a finite population's median. These estimators blend unique metrics like quartile deviation and interquartile range, enhancing data distribution analysis and outlier exclusion through robust scaling, employing decile means, the Hodges-Lehmann estimator, and tri-mean for reliable median estimation. The tri-mean proposed by^47^, the Hodges-Lehmann estimator proposed by^48^ and the decile means proposed by^49^ are the three robust metrics we used in this study. For further information about these robust measures, readers can see^50^ and^51^ for details.

Motivated by^52^, we develop a neutrosophic generalized ratio-type estimator of finite population median as

23 $$T_{i(d)N} = \hat{M}_{yN} \left[ {\left\{ {m_{1N} \left( {\frac{{\psi_{N} \hat{M}_{xN} + \delta_{N} }}{{\psi_{N} M_{xN} + \delta_{N} }}} \right)^{{\alpha_{3} }} \exp \left( {\frac{{M_{xN} - \hat{M}_{xN} }}{{M_{xN} + \hat{M}_{xN} }}} \right)} \right\} + \left\{ {m_{2N} \left( {\frac{{\psi_{N} M_{xN} + \delta_{N} }}{{\psi_{N} \hat{M}_{xN} + \delta_{N} }}} \right)^{{\alpha_{4} }} } \right\}} \right],$$

where $T_{i(d)N} \in [T_{i(d)L} , \, T_{i(d)U} ],$ $m_{1N} \in [m_{1L} , \, m_{1U} ]$ and $m_{2N} \in [m_{2L} , \, m_{2U} ]$ are suitable neutrosophic constants, where $\alpha_{3} {\text{ and }}\alpha_{4}$ take the values $1,\,\, - 1,\,\,2,\,\, - 2$ for developing new estimators.

*Note* $\psi_{N} \in [\psi_{L} , \, \psi_{U} ]$ and $\delta_{N} \in [\delta_{L} , \, \delta_{U} ]$ are neutrosophic functions of the known robust and non-conventional measures related to the variable $X_{N}$. Robust measures associated with $X_{N}$ are:

(i) Tri-mean:$$T_{MN} = (Q_{1N} + 2Q_{2N} + Q_{3N} )/4$$, $$T_{MN} \in [T_{ML} , \, T_{MU} ]$$
(ii) Hodges–Lehman: $H_{LN} = Median((x_{jN} + x_{kN} )/2),$ $1 \le j \le k \le N,$ $H_{LN} \in [H_{LL} , \, H_{LU} ]$
(iii) Decile mean: $D_{MN} = \sum\limits_{i = 1}^{9} {D_{iN} /9} ,$ $D_{MN} \in [D_{ML} , \, D_{MU} ]$

The non-conventional measures (i.e., interquartile range, midrange, quartile average and quartile deviation) of the supplementary variable are as follows:

(iv) Interquartile range: $Q_{RN} = Q_{3N} - Q_{1N}$, $Q_{RN} \in [Q_{RL} , \, Q_{RU} ]$
(v) Midrange:$$M_{RN} = ((x_{(1)N} + x_{(N)N} )/2)$$, $$M_{RN} \in [M_{RL} ,M_{RU} ]$$
(vi) Quartile average:$Q_{AN} = (Q_{3N} + Q_{1N} )/2$, $Q_{AN} \in [Q_{AL} , \, Q_{AU} ]$
(vii) Quartile deviation $$Q_{DN} = (Q_{3N} - Q_{1N} )/2,$$$$Q_{DN} \in [Q_{DL} , \, Q_{DU} ]$$

where $Q_{1N} \in [Q_{1L} , \, Q_{1U} ]$, $Q_{2N} \in [Q_{2L} , \, Q_{2U} ]$ and $Q_{3N} \in [Q_{3L} , \, Q_{3U} ]$ are the neutrosophic first, second and third quartiles, respectively and $D_{iN} \in [D_{iL} , \, D_{iU} ]$ is the neutrosophic decile.

By putting different values of $\alpha_{i} \, ({\text{for }}i = 3,4)$ into (23), we get the following families of estimators as.

- i. At $\alpha_{3} = 1$ and $\alpha_{4} = 2$, the proposed family of estimators reduces to
  24 $$T_{{i(d)N}}^{{ \ominus }} = \hat{M}_{{yN}} \left[ {\left\{ {m_{{1N}} \left( {\frac{{\psi _{N} \hat{M}_{{xN}} + \delta _{N} }}{{\psi _{N} M_{{xN}} + \delta _{N} }}} \right)\exp \left( {\frac{{M_{{xN}} - \hat{M}_{{xN}} }}{{M_{{xN}} + \hat{M}_{{xN}} }}} \right)} \right\} + \left\{ {m_{{2N}} \left( {\frac{{\psi _{N} M_{{xN}} + \delta _{N} }}{{\psi _{N} \hat{M}_{{xN}} + \delta _{N} }}} \right)^{2} } \right\}} \right].$$
- ii. At $\alpha_{3} = - 1$ and $\alpha_{4} = - 1;$ the proposed family of estimators reduces to
  25 $$T_{{i(d)N}}^{ \oplus } = \hat{M}_{{yN}} \left[ {\left\{ {m_{{1N}} \left( {\frac{{\psi _{N} M_{{xN}} + \delta _{N} }}{{\psi _{N} \hat{M}_{{xN}} + \delta _{N} }}} \right)\exp \left( {\frac{{M_{{xN}} - \hat{M}_{{xN}} }}{{M_{{xN}} + \hat{M}_{{xN}} }}} \right)} \right\} + \left\{ {m_{{2N}} \left( {\frac{{\psi _{N} \hat{M}_{{xN}} + \delta _{N} }}{{\psi _{N} M_{{xN}} + \delta _{N} }}} \right)} \right\}} \right].$$
- iii. At $\alpha_{3} = - 1$ and $\alpha_{4} = - 2$, the proposed family of estimators becomes
  26 $$T_{{i(d)N}}^{ \otimes } = \hat{M}_{{yN}} \left[ {\left\{ {m_{{1N}} \left( {\frac{{\psi _{N} M_{{xN}} + \delta _{N} }}{{\psi _{N} \hat{M}_{{xN}} + \delta _{N} }}} \right)\exp \left( {\frac{{M_{{xN}} - \hat{M}_{{xN}} }}{{M_{{xN}} + \hat{M}_{{xN}} }}} \right)} \right\} + \left\{ {m_{{2N}} \left( {\frac{{\psi _{N} \hat{M}_{{xN}} + \delta _{N} }}{{\psi _{N} M_{{xN}} + \delta _{N} }}} \right)^{2} } \right\}} \right].$$
- iv. At $\alpha_{3} = 2$ and $\alpha_{4} = 2$, the proposed family of estimators reduces to
  27 $$T_{{i(d)N}}^{{ \circledast }} = \hat{M}_{{yN}} \left[ {\left\{ {m_{{1N}} \left( {\frac{{\psi _{N} \hat{M}_{{xN}} + \delta _{N} }}{{\psi _{N} M_{{xN}} + \delta _{N} }}} \right)^{2} \exp \left( {\frac{{M_{{xN}} - \hat{M}_{{xN}} }}{{M_{{xN}} + \hat{M}_{{xN}} }}} \right)} \right\} + \left\{ {m_{{2N}} \left( {\frac{{\psi _{N} M_{{xN}} + \delta _{N} }}{{\psi _{N} \hat{M}_{{xN}} + \delta _{N} }}} \right)^{2} } \right\}} \right].$$
- v. At $\alpha_{3} = - 2$ and $\alpha_{4} = - 1$, the proposed family of estimators reduces to
  28 $$T_{{i(d)N}}^{{ \circledcirc }} = \hat{M}_{{yN}} \left[ {\left\{ {m_{{1N}} \left( {\frac{{\psi _{N} M_{{xN}} + \delta _{N} }}{{\psi _{N} \hat{M}_{{xN}} + \delta _{N} }}} \right)^{2} \exp \left( {\frac{{M_{{xN}} - \hat{M}_{{xN}} }}{{M_{{xN}} + \hat{M}_{{xN}} }}} \right)} \right\} + \left\{ {m_{{2N}} \left( {\frac{{\psi _{N} \hat{M}_{{xN}} + \delta _{N} }}{{\psi _{N} M_{{xN}} + \delta _{N} }}} \right)} \right\}} \right].$$

When we use robust measures with linear combinations of the median, quartile deviation, midrange, interquartile range, and quartile average of the supplementary variable in (23), we get different series of estimators such as $$T^{{\begin{array}{*{20}l} { \ominus } \hfill \\ \end{array} }}_{i(d)N}$$, $$T^{{\begin{array}{*{20}l} {\begin{array}{*{20}l} \oplus \hfill \\ \end{array} } \hfill \\ \end{array} }}_{i(d)N}$$, $$T^{{\begin{array}{*{20}l} {\begin{array}{*{20}l} \otimes \hfill \\ \end{array} } \hfill \\ \end{array} }}_{i(d)N}$$, $$T^{{\begin{array}{*{20}l} {\begin{array}{*{20}l} { \circledast } \hfill \\ \end{array} } \hfill \\ \end{array} }}_{i(d)N}$$ and $$T^{{\begin{array}{*{20}l} {\begin{array}{*{20}l} {\begin{array}{*{20}l} { \circledcirc } \hfill \\ \end{array} } \hfill \\ \end{array} } \hfill \\ \end{array} }}_{i(d)N} .$$ Few members of the family of estimators $$T^{{\begin{array}{*{20}l} {\begin{array}{*{20}l} {\begin{array}{*{20}l} { \circledcirc } \hfill \\ \end{array} } \hfill \\ \end{array} } \hfill \\ \end{array} }}_{i(d)N}$$ are given in Table 2. Putting the same values of $\psi_{N}$ and $\delta_{N}$ in $$T^{{\begin{array}{*{20}l} { \ominus } \hfill \\ \end{array} }}_{i(d)N}$$, $$T^{{\begin{array}{*{20}l} {\begin{array}{*{20}l} \oplus \hfill \\ \end{array} } \hfill \\ \end{array} }}_{i(d)N}$$, $$T^{{\begin{array}{*{20}l} {\begin{array}{*{20}l} \otimes \hfill \\ \end{array} } \hfill \\ \end{array} }}_{i(d)N}$$, $$T^{{\begin{array}{*{20}l} {\begin{array}{*{20}l} { \circledast } \hfill \\ \end{array} } \hfill \\ \end{array} }}_{i(d)N}$$ and $$T^{{\begin{array}{*{20}l} {\begin{array}{*{20}l} {\begin{array}{*{20}l} { \circledcirc } \hfill \\ \end{array} } \hfill \\ \end{array} } \hfill \\ \end{array} }}_{i(d)N}$$, we obtain several estimators.

**Table 2:** Some members of $$T^{{\begin{array}{*{20}l} {\begin{array}{*{20}l} {\begin{array}{*{20}l} { \circledcirc } \hfill \\ \end{array} } \hfill \\ \end{array} } \hfill \\ \end{array} }}_{i(d)N}$$.

$\psi_{N}$	$\delta_{N}$	$i$	$$T^{{\begin{array}{*{20}l} {\begin{array}{*{20}l} {\begin{array}{*{20}l} { \circledcirc } \hfill \\ \end{array} } \hfill \\ \end{array} } \hfill \\ \end{array} }}_{i(d)N} = \hat{M}_{yN} \left[ {m_{1N} \left( {\frac{{\psi_{N} M_{xN} + \delta_{N} }}{{\psi_{N} \hat{M}_{xN} + \delta_{N} }}} \right)^{2} \exp \left( {\frac{{M_{xN} - \hat{M}_{xN} }}{{M_{xN} + \hat{M}_{xN} }}} \right) + m_{2N} \left( {\frac{{\psi_{N} \hat{M}_{xN} + \delta_{N} }}{{\psi_{N} M_{xN} + \delta_{N} }}} \right)} \right]$$
$Q_{AN}$	$T_{MN}$	1	$$T^{{\begin{array}{*{20}l} {\begin{array}{*{20}l} {\begin{array}{*{20}l} { \circledcirc } \hfill \\ \end{array} } \hfill \\ \end{array} } \hfill \\ \end{array} }}_{1(d)N} = \hat{M}_{yN} \left[ {m_{1N} \left( {\frac{{Q_{AN} M_{xN} + T_{MN} }}{{Q_{AN} \hat{M}_{xN} + T_{MN} }}} \right)^{2} \exp \left( {\frac{{M_{xN} - \hat{M}_{xN} }}{{M_{xN} + \hat{M}_{xN} }}} \right) + m_{2N} \left( {\frac{{Q_{AN} \hat{M}_{xN} + T_{MN} }}{{Q_{AN} M_{xN} + T_{MN} }}} \right)} \right]$$
$M_{RN}$	$T_{MN}$	2	$$T^{{\begin{array}{*{20}l} {\begin{array}{*{20}l} {\begin{array}{*{20}l} { \circledcirc } \hfill \\ \end{array} } \hfill \\ \end{array} } \hfill \\ \end{array} }}_{2(d)N} = \hat{M}_{yN} \left[ {m_{1N} \left( {\frac{{M_{RN} M_{xN} + T_{MN} }}{{M_{RN} \hat{M}_{xN} + T_{MN} }}} \right)^{2} \exp \left( {\frac{{M_{xN} - \hat{M}_{xN} }}{{M_{xN} + \hat{M}_{xN} }}} \right) + m_{2N} \left( {\frac{{M_{RN} \hat{M}_{xN} + T_{MN} }}{{M_{RN} M_{xN} + T_{MN} }}} \right)} \right]$$
$H_{LN}$	$T_{MN}$	3	$$T^{{\begin{array}{*{20}l} {\begin{array}{*{20}l} {\begin{array}{*{20}l} { \circledcirc } \hfill \\ \end{array} } \hfill \\ \end{array} } \hfill \\ \end{array} }}_{3(d)N} = \hat{M}_{yN} \left[ {m_{1N} \left( {\frac{{H_{LN} M_{xN} + T_{MN} }}{{H_{LN} \hat{M}_{xN} + T_{MN} }}} \right)^{2} \exp \left( {\frac{{M_{xN} - \hat{M}_{xN} }}{{M_{xN} + \hat{M}_{xN} }}} \right) + m_{2N} \left( {\frac{{H_{LN} \hat{M}_{xN} + T_{MN} }}{{H_{LN} M_{xN} + T_{MN} }}} \right)} \right]$$
$T_{MN}$	$H_{LN}$	4	$$T^{{\begin{array}{*{20}l} {\begin{array}{*{20}l} {\begin{array}{*{20}l} { \circledcirc } \hfill \\ \end{array} } \hfill \\ \end{array} } \hfill \\ \end{array} }}_{4(d)N} = \hat{M}_{yN} \left[ {m_{1N} \left( {\frac{{T_{MN} M_{xN} + H_{LN} }}{{T_{MN} \hat{M}_{xN} + H_{LN} }}} \right)^{2} \exp \left( {\frac{{M_{xN} - \hat{M}_{xN} }}{{M_{xN} + \hat{M}_{xN} }}} \right) + m_{2N} \left( {\frac{{T_{MN} \hat{M}_{xN} + H_{LN} }}{{T_{MN} M_{xN} + H_{LN} }}} \right)} \right]$$
$Q_{AN}$	$H_{LN}$	5	$$T^{{\begin{array}{*{20}l} {\begin{array}{*{20}l} {\begin{array}{*{20}l} { \circledcirc } \hfill \\ \end{array} } \hfill \\ \end{array} } \hfill \\ \end{array} }}_{5(d)N} = \hat{M}_{yN} \left[ {m_{1N} \left( {\frac{{Q_{AN} M_{xN} + H_{LN} }}{{Q_{AN} \hat{M}_{xN} + H_{LN} }}} \right)^{2} \exp \left( {\frac{{M_{xN} - \hat{M}_{xN} }}{{M_{xN} + \hat{M}_{xN} }}} \right) + m_{2N} \left( {\frac{{Q_{AN} \hat{M}_{xN} + H_{LN} }}{{Q_{AN} M_{xN} + H_{LN} }}} \right)} \right]$$
$H_{LN}$	$Q_{RN}$	6	$$T^{{\begin{array}{*{20}l} {\begin{array}{*{20}l} {\begin{array}{*{20}l} { \circledcirc } \hfill \\ \end{array} } \hfill \\ \end{array} } \hfill \\ \end{array} }}_{6(d)N} = \hat{M}_{yN} \left[ {m_{1N} \left( {\frac{{H_{LN} M_{xN} + Q_{RN} }}{{H_{LN} \hat{M}_{xN} + Q_{RN} }}} \right)^{2} \exp \left( {\frac{{M_{xN} - \hat{M}_{xN} }}{{M_{xN} + \hat{M}_{xN} }}} \right) + m_{2N} \left( {\frac{{H_{LN} \hat{M}_{xN} + Q_{RN} }}{{H_{LN} M_{xN} + Q_{RN} }}} \right)} \right]$$
$M_{RN}$	$H_{LN}$	7	$$T^{{\begin{array}{*{20}l} {\begin{array}{*{20}l} {\begin{array}{*{20}l} { \circledcirc } \hfill \\ \end{array} } \hfill \\ \end{array} } \hfill \\ \end{array} }}_{7(d)N} = \hat{M}_{yN} \left[ {m_{1N} \left( {\frac{{M_{RN} M_{xN} + H_{LN} }}{{M_{RN} \hat{M}_{xN} + H_{LN} }}} \right)^{2} \exp \left( {\frac{{M_{xN} - \hat{M}_{xN} }}{{M_{xN} + \hat{M}_{xN} }}} \right) + m_{2N} \left( {\frac{{M_{RN} \hat{M}_{xN} + H_{LN} }}{{M_{RN} M_{xN} + H_{LN} }}} \right)} \right]$$
$H_{LN}$	$D_{MN}$	8	$$T^{{\begin{array}{*{20}l} {\begin{array}{*{20}l} {\begin{array}{*{20}l} { \circledcirc } \hfill \\ \end{array} } \hfill \\ \end{array} } \hfill \\ \end{array} }}_{8(d)N} = \hat{M}_{yN} \left[ {m_{1N} \left( {\frac{{H_{LN} M_{xN} + D_{MN} }}{{H_{LN} \hat{M}_{xN} + D_{MN} }}} \right)^{2} \exp \left( {\frac{{M_{xN} - \hat{M}_{xN} }}{{M_{xN} + \hat{M}_{xN} }}} \right) + m_{2N} \left( {\frac{{H_{LN} \hat{M}_{xN} + D_{MN} }}{{H_{LN} M_{xN} + D_{MN} }}} \right)} \right]$$
$D_{MN}$	$T_{MN}$	9	$$T^{{\begin{array}{*{20}l} {\begin{array}{*{20}l} {\begin{array}{*{20}l} { \circledcirc } \hfill \\ \end{array} } \hfill \\ \end{array} } \hfill \\ \end{array} }}_{9(d)N} = \hat{M}_{yN} \left[ {m_{1N} \left( {\frac{{D_{MN} M_{xN} + T_{MN} }}{{D_{MN} \hat{M}_{xN} + T_{MN} }}} \right)^{2} \exp \left( {\frac{{M_{xN} - \hat{M}_{xN} }}{{M_{xN} + \hat{M}_{xN} }}} \right) + m_{2N} \left( {\frac{{D_{MN} \hat{M}_{xN} + T_{MN} }}{{D_{MN} M_{xN} + T_{MN} }}} \right)} \right]$$
$M_{RN}$	$D_{MN}$	10	$$T^{{\begin{array}{*{20}l} {\begin{array}{*{20}l} {\begin{array}{*{20}l} { \circledcirc } \hfill \\ \end{array} } \hfill \\ \end{array} } \hfill \\ \end{array} }}_{10(d)N} = \hat{M}_{yN} \left[ {m_{1N} \left( {\frac{{M_{RN} M_{xN} + D_{MN} }}{{M_{RN} \hat{M}_{xN} + D_{MN} }}} \right)^{2} \exp \left( {\frac{{M_{xN} - \hat{M}_{xN} }}{{M_{xN} + \hat{M}_{xN} }}} \right) + m_{2N} \left( {\frac{{M_{RN} \hat{M}_{xN} + D_{MN} }}{{M_{RN} M_{xN} + D_{MN} }}} \right)} \right]$$
$Q_{AN}$	$D_{MN}$	11	$$T^{{\begin{array}{*{20}l} {\begin{array}{*{20}l} {\begin{array}{*{20}l} { \circledcirc } \hfill \\ \end{array} } \hfill \\ \end{array} } \hfill \\ \end{array} }}_{11(d)N} = \hat{M}_{yN} \left[ {m_{1N} \left( {\frac{{Q_{AN} M_{xN} + D_{MN} }}{{Q_{AN} \hat{M}_{xN} + D_{MN} }}} \right)^{2} \exp \left( {\frac{{M_{xN} - \hat{M}_{xN} }}{{M_{xN} + \hat{M}_{xN} }}} \right) + m_{2N} \left( {\frac{{Q_{AN} \hat{M}_{xN} + D_{MN} }}{{Q_{AN} M_{xN} + D_{MN} }}} \right)} \right]$$
$D_{MN}$	$Q_{AN}$	12	$$T^{{\begin{array}{*{20}l} {\begin{array}{*{20}l} {\begin{array}{*{20}l} { \circledcirc } \hfill \\ \end{array} } \hfill \\ \end{array} } \hfill \\ \end{array} }}_{12(d)N} = \hat{M}_{yN} \left[ {m_{1N} \left( {\frac{{D_{MN} M_{xN} + Q_{AN} }}{{D_{MN} \hat{M}_{xN} + Q_{AN} }}} \right)^{2} \exp \left( {\frac{{M_{xN} - \hat{M}_{xN} }}{{M_{xN} + \hat{M}_{xN} }}} \right) + m_{2N} \left( {\frac{{D_{MN} \hat{M}_{xN} + Q_{AN} }}{{D_{MN} M_{xN} + Q_{AN} }}} \right)} \right]$$

We can obtain several optimal estimators by placing suitable constants or known conventional parameters of the supplementary variable in place of $\psi_{N}$ and $\delta_{N}$ into (23). Conventional parameters related to the supplementary variable $X_{N}$ are variance, standard deviation, coefficient of variation, coefficient of skewness, coefficient of kurtosis, coefficient of correlation, and so forth.

Bias, MSE, and minimum MSE of the proposed neutrosophic generalized family of estimators $T_{i(d)N}$ in terms of $e_{oN}$ and $e_{1N}$ are expressed as,

29 $$Bias(T_{i(d)N} ) = M_{yN} \left[ {m_{1N} \left( {1 + \frac{{\lambda_{N} C_{{M_{xN} }}^{2} }}{2}\left( {\frac{3}{4} - \alpha_{3} \theta_{N} + \alpha_{3} (\alpha_{3} - 1)\theta_{N}^{2} } \right) + \lambda_{N} \rho_{N} C_{{M_{yN} }} C_{{M_{xN} }} \left( {\alpha_{3} \theta_{N} - \frac{1}{2}} \right)} \right)} \right.\left. { + m_{2N} \left( {1 + \frac{{\alpha_{4} (\alpha_{4} + 1)\theta_{N}^{2} \lambda_{N} C_{{M_{xN} }}^{2} }}{2} - \alpha_{4} \theta_{N} \lambda_{N} \rho_{N} C_{{M_{yN} }} C_{{M_{xN} }} } \right) - 1} \right]$$

where $$\theta_{N} = \psi_{N} M_{xN} /(\psi_{N} M_{xN} + \delta_{N} )$$.

The MSE of suggested estimator up to the first order of approximation as

30 $$MSE(T_{i(d)N} ) \cong M_{yN}^{2} [1 + m_{1N}^{2} A_{1N} + m_{2N}^{2} A_{2N} + 2m_{1N} m_{2N} A_{3N} - 2m_{1N} A_{4N} - 2m_{2N} A_{5N} ],$$

where $$A_{1N} = \left[ {1 + \lambda_{N} \left\{ {C_{{M_{yN} }}^{2} + C_{{M_{xN} }}^{2} (1 + 2\alpha_{3}^{2} \theta_{N}^{2} - \alpha_{3} \theta_{N}^{2} - 2\alpha_{3} \theta_{N} ) - 2\rho_{N} C_{{M_{yN} }} C_{{M_{xN} }} (1 - 2\alpha_{3} \theta_{N} )} \right\}} \right]$$

$$A_{2N} = [1 + \lambda_{N} \{ C_{{M_{yN} }}^{2} + \theta_{N}^{2} C_{{M_{xN} }}^{2} (2\alpha_{4}^{2} + \alpha_{4} ) - 4\alpha_{4} \theta_{N} \rho_{N} C_{{M_{yN} }} C_{{M_{xN} }} \} ],$$

$$A_{3N} = \left[ {1 + \lambda_{N} \left\{ {C_{{M_{yN} }}^{2} - \rho_{N} C_{{M_{yN} }} C_{{M_{xN} }} (2\theta_{N} (\alpha_{4} - \alpha_{3} ) + 1) - C_{{M_{xN} }}^{2} \left( {\alpha_{3} \alpha_{4} \theta_{N}^{2} - \frac{3}{8} + \frac{{\alpha_{3} \theta_{N} }}{2}} \right.} \right.} \right.$$

$$\, \left. {\left. {\left. { - \frac{{\alpha_{4} \theta_{N} }}{2} - \frac{{\alpha_{4} (\alpha_{4} + 1)\theta_{N}^{2} }}{2} - \frac{1}{2}\alpha_{3} (\alpha_{3} - 1)\theta_{N}^{2} } \right)} \right\}} \right]$$

$$A_{4N} = \left[ {1 + \lambda_{N} \rho_{N} C_{{M_{yN} }} C_{{M_{xN} }} \left( {\alpha_{3} \theta_{N} - \frac{1}{2}} \right) + \lambda_{N} \frac{{C_{{M_{xN} }}^{2} }}{2}\left( {\frac{3}{4} - \alpha_{3} \theta_{N} + \alpha_{3} (\alpha_{3} - 1)\theta_{N}^{2} } \right)} \right]$$ and $$A_{5N} = \left[ {1 + \frac{1}{2}\alpha_{4} (\alpha_{4} + 1)\theta_{N}^{2} \lambda_{N} C_{{M_{xN} }}^{2} - \alpha_{4} \theta_{N} \lambda_{N} \rho_{N} C_{{M_{yN} }} C_{{M_{xN} }} } \right].$$

The minimum MSE at the optimum values $m_{1N(opt)} = \frac{{(A_{2N} A_{4N} - A_{3N} A_{5N} )}}{{(A_{1N} A_{2N} - A_{3N}^{2} )}}$ and $m_{2N(opt)} = \frac{{(A_{1N} A_{5N} - A_{3N} A_{4N} )}}{{(A_{1N} A_{2N} - A_{3N}^{2} )}},$ is given by

31 $$MSE(T_{i(d)N} )_{\min } \cong M_{yN}^{2} \left[ {1 - \frac{{(A_{2N} A_{4N}^{2} + A_{1N} A_{5N}^{2} - 2A_{3N} A_{4N} A_{5N} )}}{{(A_{1N} A_{2N} - A_{3N}^{2} )}}} \right]$$

### Efficiency comparison

(i). By comparing (2) and (23), $Var(\hat{M}_{0N} ) > MSE(T_{i(d)N} )_{\min }$ if $\left[ {\Theta_{2N} \left( {\lambda_{N} M_{yN}^{2} C_{{M_{yN} }}^{2} - 1} \right) + \Theta_{1N} } \right]\, > 0$, where $\Theta_{1N} = A_{2N} A_{4N}^{2} + A_{1N} A_{5N}^{2} - 2A_{3N} A_{4N} A_{5N}$ and $\Theta_{2N} = A_{1N} A_{2N} - A_{3N}^{2}$.

(ii) By comparing (5) and (23), $MSE(\hat{M}_{RN} ) > MSE(T_{i(d)N} )_{\min }$ if

$$\left[ {\Theta_{2N} \left( {\lambda_{hN} C_{{M_{yN} }}^{2} + \lambda_{N} C_{{M_{xN} }}^{2} - 2\lambda_{N} C_{{M_{yxN} }} - 1} \right) + \Theta_{1N} } \right]\, > 0$$

(iii) By comparing (8) and (23), $MSE(\hat{M}_{EN} ) > MSE(T_{i(d)N} )_{\min }$ if

$$\left[ {\Theta_{2N} \left( {\lambda_{N} C_{{M_{yN} }}^{2} + \frac{1}{4}\lambda_{N} C_{{M_{xN} }}^{2} - \lambda_{N} C_{{M_{yxN} }} - 1} \right) + \Theta_{1N} } \right]\, > 0$$

(iv) By comparing (10) and (23), $Var(\hat{M}_{{D_{0} N}} )_{\min } > MSE(T_{i(d)N} )_{\min }$ if

$$\left[ {C_{{M_{yN} }}^{2} \lambda_{N} \left( {1 - \rho_{yxN}^{2} } \right) - 1 + \frac{{\Theta_{1N} }}{{\Theta_{2N} }}} \right] \, > 0$$

(v) By comparing (19) and (23), $MSE(\hat{M}_{{D_{1} N}} )_{\min } > MSE(T_{i(d)N} )_{\min }$ if

$$\left[ {\frac{{\Theta_{1N} }}{{\Theta_{2N} }} - \frac{{B_{0N} }}{{A_{0N} B_{0N} - C_{0N}^{2} + B_{0N} }}} \right]\, > 0$$

(vi) By comparing (20) and (23), $MSE(\hat{M}_{{D_{2} N}} )_{\min } > MSE(T_{i(d)N} )_{\min }$ if

$$\left[ {\frac{{\Theta_{1N} }}{{\Theta_{2N} }} - \frac{{\left( {A_{1N} B_{1N}^{2} + B_{1N} C_{1N}^{2} - 2B_{1N} C_{1N} D_{1N} + B_{1N}^{2} + 2B_{1N} C_{1N} - 2B_{1N} D_{1N} + B_{1N} } \right)}}{{A_{1N} B_{1N} - D_{1N}^{2} + B_{1N} }}} \right]\, > 0$$

(vii) By comparing (21) and (23), $\,MSE(\hat{M}_{{D_{3} N}} )_{\min } > MSE(T_{i(d)N} )_{\min }$ if

$$\left[ {\frac{{\Theta_{1N} }}{{\Theta_{2N} }} - \frac{{A_{2N} D_{2N}^{2} + B_{2N} C_{2N}^{2} - 2C_{2N} D_{2N} E_{2N} + 2B_{2N} C_{2N} + D_{2N}^{2} - 2D_{2N} E_{2N} + B_{2N} }}{{A_{2N} B_{2N} - E_{2N}^{2} + B_{2N} }}} \right]\, > 0$$

(vii) By comparing (22) and (23), $MSE(\hat{M}_{{D_{4} N}} )_{\min } > MSE(T_{i(d)N} )_{\min }$ if

$$\left[ {\frac{{\Theta_{1N} }}{{\Theta_{2N} }} - \frac{{A_{3N} B_{3N}^{2} + B_{3N} C_{3N}^{2} - 2B_{3N} C_{3N} D_{3N} + B_{3N}^{2} + 2B_{3N} C_{3N} - 2B_{3N} D_{3N} + B_{3N} }}{{A_{3N} B_{3N} - D_{3N}^{2} + B_{3N} }}} \right]\, > 0$$

Hence, robustness is evaluated in this case to compare the proposed neutrosophic generalized estimators with other neutrosophic estimators in (1), (3), (6), (9), (11), (12), (13), and (14) to find the more effective neutrosophic median estimator. Additionally, we use real-world datasets to determine the relative effectiveness of different estimators.

## Real-life application

In terms of relative efficiency, we compare the suggested family of estimators' performance to that of other competitive estimators. We chose two real-world indeterminacy interval datasets for this purpose.

Regarding relative efficiency, we compare the suggested family of estimators' performance to that of other competitive estimators. For this purpose, we chose two real-world indeterminacy interval datasets.

The first one is the Daily stock price, which is used as a neutrosophic variable because, on each day, a stock's price fluctuates between an opening price (the price during which trade begins) and a closing price (the price at which trade stops for the day). The price constantly fluctuates between a high (the highest price of the day) and a low (the lowest price), which may or may not be similar to the opening or closing price. We estimate the high and low price intervals within which the stock price falls by utilizing the daily starting price as a supplementary variable that is not a neutrosophic variable since its value is set and known for each day.

*Population I* Source: ^53^ (1^st^ June 2022 to 29^th^ July 2022) from the link: https://finance.yahoo.com/quote/SMSN.IL/history?p=SMSN.IL. $Y_{N} =$ Low & High prices; $X_{N} =$ Opening price.

*Population II* Source: ^54^ (1^st^ Feb 2022 to 29^th^ July 2022) from the link: https://finance.yahoo.com/quote/SZKMY/history?p=SZKMY. $Y_{N} =$ Low & High prices; $X_{N} =$ Opening price, where $Y_{N} \in (Y_{L} ,Y_{U} )$ corresponds to the independent determinate variable $X_{N} \in (X_{L} ,X_{U} )$.

**Figure 2:**
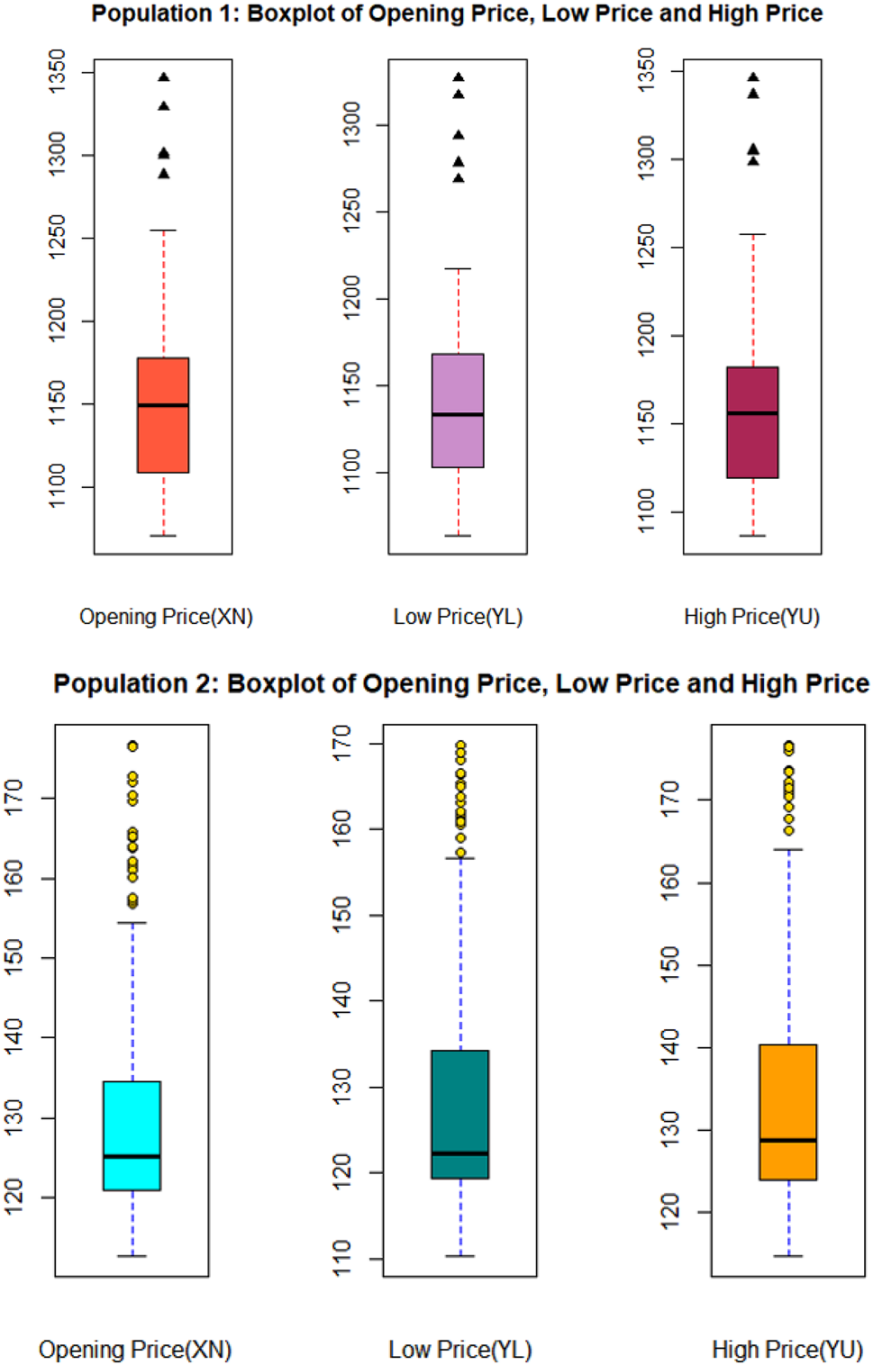
Boxplot of Populations I and II for each variable.

**Figure 3:**
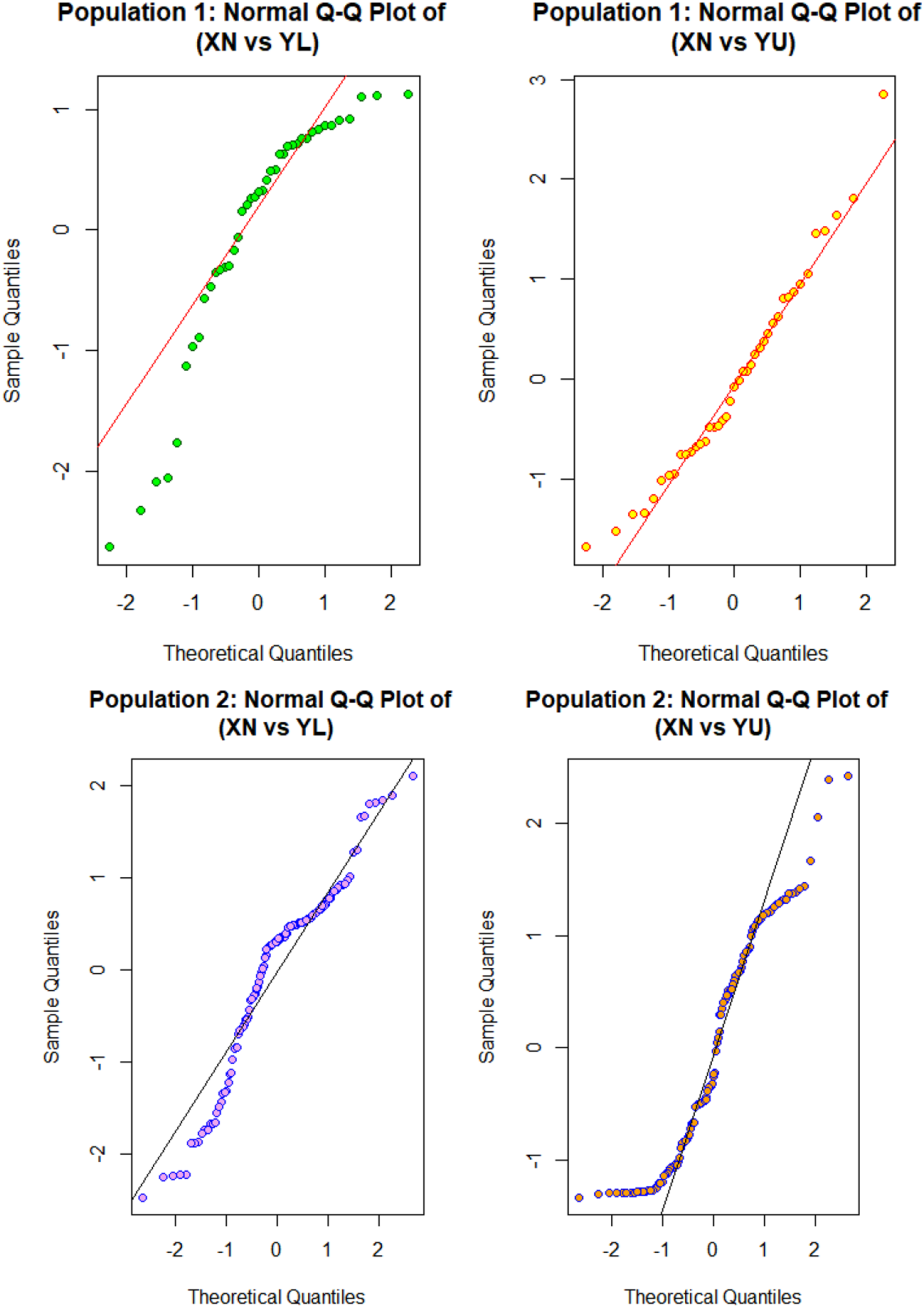
Normal Q-Q plot of Populations I and II for variable $X_{N}$ and $Y_{N}$.

Figures 2 and 3 show the trend of real-world data sets using box plots, which aids in displaying the skewness of the data. The minimum score (Lower Fence), the lower quartile, the median, the upper quartile, and the maximum score summarise data using boxplots (Upper Fence). As shown in Fig. 2, our positively skewed data suggests that the median is closer to the lower quartile. The boxplot with points outside the whiskers shows a few outliers in the data.

Figure 3 elaborates on the pivotal role of Q-Q (quantile–quantile) plots in statistics. These plots facilitate the graphical comparison of two probability distributions through their quantiles. They are instrumental in determining the distribution type of a random variable, spotting outliers, and assessing skewness. By plotting theoretical quantiles against sample quantiles, Q-Q plots reveal distribution traits, including skewness. Notably, deviations in the plot’s upper end and a pronounced right tail indicate a right-skewed distribution, as demonstrated in standard Q-Q plot interpretations.

In addition, the following formulae are used to get the percentage relative efficiency (PRE) $PRE(\hat{M}_{0N} ,\hat{M}_{iN} ) = \frac{{Var(\hat{M}_{0N} )}}{{MSE(\hat{M}_{iN} )}} \times 100,$ $({\text{for }}i = 0, \, R, \, E, \, D_{0} , \, D_{1} , \, D_{2} , \, D_{3} {\text{ and }}D_{4} )$ and $PRE(\hat{M}_{0N} ,T_{i(d)N} ) = \frac{{Var(\hat{M}_{0N} )}}{{MSE(T_{i(d)N} )}} \times 100,$ $({\text{for }}i = 1,...,12)$.

Table 3 presents the complete descriptions of each population mentioned below. Table 4 presents the complete descriptions of each population mentioned below. Tables 5, 6, 7, 8, 9 and 10 elaborate the PREs of all neutrosophic estimators relative to $\hat{M}_{0N}$. It is observed that the PREs of $T_{i(d)N}$ estimators change with the choices of $\alpha_{3}$ and $\alpha_{4}$. It is further noted that the performance of $T_{i(d)N}$ is the best among all the estimators proposed here.

**Table 3:** Descriptive statistics of populations for single auxiliary variable.

Values	Population I	Population II
$N$	41	124
$n_{N}$	[8, 8]	[45, 45]
$M_{yN}$	[1133, 1156.5]	[122.2155, 128.775]
$M_{xN}$	[1149.5, 1149.5]	[125.103, 125.103]
$f_{yN} (M_{yN} )$	[0.005798982, 0.005795176]	[0.02228004, 0.0214323]
$f_{xN} (M_{xN} )$	[0.005660414, 0.005660414]	[0.02137693, 0.02137693]
$\rho_{yxN}$	[0.9512195, 0.9512195]	[0.6774194, 0.6774194]
$C_{{M_{yN} }}$	[0.1522013, 0.1492065]	[0.3672466, 0.3623261]
$C_{{M_{xN} }}$	[0.153689, 0.153689]	[0.3739271, 0.3739271]
$M_{RN}$	[1208.75, 1208.75]	[144.6945, 144.6945]
$Q_{DN}$	[34.5, 34.5]	[6.769003, 6.769003]
$Q_{AN}$	[1143.5, 1143.5]	[127.7385, 127.7385]
$Q_{RN}$	[69, 69]	[13.53801, 13.53801]
$T_{MN}$	[1146.5, 1146.5]	[126.4208, 126.4208]
$H_{LN}$	[1142.625, 1142.625]	[124.565, 124.565]
$D_{MN}$	[1156.778, 1156.778]	[130.1744, 130.1744]

**Table 4:** PRE’s of proposed neutrosophic estimators to $\hat{M}_{0N}$.

Estimators	Population I	Population II
$\hat{M}_{0N}$	[100,100]	[100, 100]
$\hat{M}_{RN}$	[1014.094, 986.2470]	[152.1541, 149.9603]
$\hat{M}_{EN}$	[339.6810, 350.3235]	[175.6124, 176.3182]
$\hat{M}_{{D_{0N} }}$	[1050.625, 1050.625]	[184.8077, 184.8077]
$\hat{M}_{{D_{1N} }}$	[1050.683, 1050.681]	[184.8554, 184.8542]
$\hat{M}_{{D_{2N} }}$	[1050.683, 1050.681]	[184.8555, 184.8542]
$\hat{M}_{{D_{3N} }}$	[1050.944, 1050.946]	[184.8810, 184.8798]
$\hat{M}_{{D_{4N} }}$	[1052.984, 1053.050]	[184.9908, 184.9908]

**Table 5:** PRE’s of proposed neutrosophic estimators $$T^{{\begin{array}{*{20}l} { \ominus } \hfill \\ \end{array} }}_{i(d)N}$$ to $\hat{M}_{0N}$.

Estimators	Population I	Population II
$$T^{{\begin{array}{*{20}l} { \ominus } \hfill \\ \end{array} }}_{1(d)N}$$	[1055.841, 1056.150]	[185.1490, 185.1518]
$$T^{{\begin{array}{*{20}l} { \ominus } \hfill \\ \end{array} }}_{2(d)N}$$	[1055.842, 1056.152]	[185.1499, 185.1527]
$$T^{{\begin{array}{*{20}l} { \ominus } \hfill \\ \end{array} }}_{3(d)N}$$	[1055.841, 1056.150]	[185.1488, 185.1516]
$$T^{{\begin{array}{*{20}l} { \ominus } \hfill \\ \end{array} }}_{4(d)N}$$	[1055.841, 1056.151]	[185.1491, 185.1518]
$$T^{{\begin{array}{*{20}l} { \ominus } \hfill \\ \end{array} }}_{5(d)N}$$	[1055.841, 1056.151]	[185.1491, 185.1519]
$$T^{{\begin{array}{*{20}l} { \ominus } \hfill \\ \end{array} }}_{6(d)N}$$	[1055.864, 1056.174]	[185.1560, 185.1588]
$$T^{{\begin{array}{*{20}l} { \ominus } \hfill \\ \end{array} }}_{7(d)N}$$	[1055.842, 1056.152]	[185.1500, 185.1528]
$$T^{{\begin{array}{*{20}l} { \ominus } \hfill \\ \end{array} }}_{8(d)N}$$	[1055.841, 1056.150]	[185.1486, 185.1513]
$$T^{{\begin{array}{*{20}l} { \ominus } \hfill \\ \end{array} }}_{9(d)N}$$	[1055.841, 1056.151]	[185.1492, 185.1519]
$$T^{{\begin{array}{*{20}l} { \ominus } \hfill \\ \end{array} }}_{10(d)N}$$	[1055.842, 1056.152]	[185.1497, 185.1525]
$$T^{{\begin{array}{*{20}l} { \ominus } \hfill \\ \end{array} }}_{11(d)N}$$	[1055.841, 1056.150]	[185.1488, 185.1515]
$$T^{{\begin{array}{*{20}l} { \ominus } \hfill \\ \end{array} }}_{12(d)N}$$	[1055.841, 1056.151]	[185.1491, 185.1518]

**Table 6:** PRE’s of proposed neutrosophic estimators $T^{ \oplus }_{i(d)N}$ to $\hat{M}_{0N}$.

Estimators	Population I	Population II
$T^{ \oplus }_{1(d)N}$	[1053.554, 1053.833]	[185.1119, 185.1161]
$T^{ \oplus }_{2(d)N}$	[1053.554, 1053.833]	[185.1125, 185.1167]
$T^{ \oplus }_{3(d)N}$	[1053.554, 1053.833]	[185.1118, 185.1159]
$T^{ \oplus }_{4(d)N}$	[1053.554, 1053.833]	[185.1119, 185.1161]
$T^{ \oplus }_{5(d)N}$	[1053.554, 1053.833]	[185.1120, 185.1162]
$T^{ \oplus }_{6(d)N}$	[1053.565, 1053.844]	[185.1165, 185.1207]
$T^{ \oplus }_{7(d)N}$	[1053.555, 1053.833]	[185.1126, 185.1168]
$T^{ \oplus }_{8(d)N}$	[1053.554, 1053.832]	[185.1116, 185.1158]
$T^{ \oplus }_{9(d)N}$	[1053.554, 1053.833]	[185.1120, 185.1162]
$T^{ \oplus }_{10(d)N}$	[1053.554, 1053.833]	[185.1124, 185.1165]
$T^{ \oplus }_{11(d)N}$	[1053.554, 1053.832]	[185.1118, 185.1159]
$T^{ \oplus }_{12(d)N}$	[1053.554, 1053.833]	[185.1119, 185.1161]

**Table 7:** PRE’s of proposed neutrosophic estimators $T^{ \otimes }_{i(d)N}$ to $\hat{M}_{0N}$.

Estimators	Population I	Population II
$T^{ \otimes }_{1(d)N}$	[1056.555, 1057.150]	[185.3314, 185.3431]
$T^{ \otimes }_{2(d)N}$	[1056.557, 1057.151]	[185.3327, 185.3444]
$T^{ \otimes }_{3(d)N}$	[1056.555, 1057.150]	[185.3311, 185.3428]
$T^{ \otimes }_{4(d)N}$	[1056.556, 1057.150]	[185.3315, 185.3431]
$T^{ \otimes }_{5(d)N}$	[1056.555, 1057.150]	[185.3316, 185.3432]
$T^{ \otimes }_{6(d)N}$	[1056.579, 1057.175]	[185.3412, 185.3531]
$T^{ \otimes }_{7(d)N}$	[1056.557, 1057.151]	[185.3328, 185.3445]
$T^{ \otimes }_{8(d)N}$	[1056.555, 1057.149]	[185.3308, 185.3424]
$T^{ \otimes }_{9(d)N}$	[1056.556, 1057.150]	[185.3316, 185.3433]
$T^{ \otimes }_{10(d)N}$	[1056.557, 1057.151]	[185.3324, 185.3441]
$T^{ \otimes }_{11(d)N}$	[1056.555, 1057.149]	[185.3311, 185.3427]
$T^{ \otimes }_{12(d)N}$	[1056.556, 1057.150]	[185.3315, 185.3432]

**Table 8:** PRE’s of proposed neutrosophic estimators $$T^{{\begin{array}{*{20}l} {\begin{array}{*{20}l} { \circledast } \hfill \\ \end{array} } \hfill \\ \end{array} }}_{i(d)N}$$ to $\hat{M}_{0N}$.

Estimators	Population I	Population II
$$T^{{\begin{array}{*{20}l} {\begin{array}{*{20}l} { \circledast } \hfill \\ \end{array} } \hfill \\ \end{array} }}_{1(d)N}$$	[1064.050, 1064.946]	[185.5451, 185.5588]
$$T^{{\begin{array}{*{20}l} {\begin{array}{*{20}l} { \circledast } \hfill \\ \end{array} } \hfill \\ \end{array} }}_{2(d)N}$$	[1064.053, 1064.949]	[185.5475, 185.5613]
$$T^{{\begin{array}{*{20}l} {\begin{array}{*{20}l} { \circledast } \hfill \\ \end{array} } \hfill \\ \end{array} }}_{3(d)N}$$	[1064.050, 1064.946]	[185.5445, 185.5583]
$$T^{{\begin{array}{*{20}l} {\begin{array}{*{20}l} { \circledast } \hfill \\ \end{array} } \hfill \\ \end{array} }}_{4(d)N}$$	[1064.050, 1064.946]	[185.5451, 185.5589]
$$T^{{\begin{array}{*{20}l} {\begin{array}{*{20}l} { \circledast } \hfill \\ \end{array} } \hfill \\ \end{array} }}_{5(d)N}$$	[1064.050, 1064.946]	[185.5454, 185.5591]
$$T^{{\begin{array}{*{20}l} {\begin{array}{*{20}l} { \circledast } \hfill \\ \end{array} } \hfill \\ \end{array} }}_{6(d)N}$$	[1064.111, 1065.010]	[185.5639, 185.5781]
$$T^{{\begin{array}{*{20}l} {\begin{array}{*{20}l} { \circledast } \hfill \\ \end{array} } \hfill \\ \end{array} }}_{7(d)N}$$	[1064.053, 1064.950]	[185.5478, 185.5616]
$$T^{{\begin{array}{*{20}l} {\begin{array}{*{20}l} { \circledast } \hfill \\ \end{array} } \hfill \\ \end{array} }}_{8(d)N}$$	[1064.049, 1064.945]	[185.5439, 185.5576]
$$T^{{\begin{array}{*{20}l} {\begin{array}{*{20}l} { \circledast } \hfill \\ \end{array} } \hfill \\ \end{array} }}_{9(d)N}$$	[1064.050, 1064.946]	[185.5454, 185.5592]
$$T^{{\begin{array}{*{20}l} {\begin{array}{*{20}l} { \circledast } \hfill \\ \end{array} } \hfill \\ \end{array} }}_{10(d)N}$$	[1064.053, 1064.949]	[185.5470, 185.5608]
$$T^{{\begin{array}{*{20}l} {\begin{array}{*{20}l} { \circledast } \hfill \\ \end{array} } \hfill \\ \end{array} }}_{11(d)N}$$	[1064.049, 1064.945]	[185.5444, 185.5582]
$$T^{{\begin{array}{*{20}l} {\begin{array}{*{20}l} { \circledast } \hfill \\ \end{array} } \hfill \\ \end{array} }}_{12(d)N}$$	[1064.051, 1064.947]	[185.5452, 185.5590]

**Table 9:** PRE’s of proposed neutrosophic estimators $$T^{{\begin{array}{*{20}l} {\begin{array}{*{20}l} {\begin{array}{*{20}l} { \circledcirc } \hfill \\ \end{array} } \hfill \\ \end{array} } \hfill \\ \end{array} }}_{i(d)N}$$ to $\hat{M}_{0N}$.

Estimators	Population I	Population II
$$T^{{\begin{array}{*{20}l} {\begin{array}{*{20}l} {\begin{array}{*{20}l} { \circledcirc } \hfill \\ \end{array} } \hfill \\ \end{array} } \hfill \\ \end{array} }}_{1(d)N}$$	[1068.681, 1069.610]	[185.6116, 185.6235]
$$T^{{\begin{array}{*{20}l} {\begin{array}{*{20}l} {\begin{array}{*{20}l} { \circledcirc } \hfill \\ \end{array} } \hfill \\ \end{array} } \hfill \\ \end{array} }}_{2(d)N}$$	[1068.685, 1069.613]	[185.6135, 185.6254]
$$T^{{\begin{array}{*{20}l} {\begin{array}{*{20}l} {\begin{array}{*{20}l} { \circledcirc } \hfill \\ \end{array} } \hfill \\ \end{array} } \hfill \\ \end{array} }}_{3(d)N}$$	[1068.681, 1069.610]	[185.6112, 185.6231]
$$T^{{\begin{array}{*{20}l} {\begin{array}{*{20}l} {\begin{array}{*{20}l} { \circledcirc } \hfill \\ \end{array} } \hfill \\ \end{array} } \hfill \\ \end{array} }}_{4(d)N}$$	[1068.682, 1069.610]	[185.6117, 185.6236]
$$T^{{\begin{array}{*{20}l} {\begin{array}{*{20}l} {\begin{array}{*{20}l} { \circledcirc } \hfill \\ \end{array} } \hfill \\ \end{array} } \hfill \\ \end{array} }}_{5(d)N}$$	[1068.681, 1069.610]	[185.6118, 185.6237]
$$T^{{\begin{array}{*{20}l} {\begin{array}{*{20}l} {\begin{array}{*{20}l} { \circledcirc } \hfill \\ \end{array} } \hfill \\ \end{array} } \hfill \\ \end{array} }}_{6(d)N}$$	[1068.738, 1069.668]	[185.6263, 185.6385]
$$T^{{\begin{array}{*{20}l} {\begin{array}{*{20}l} {\begin{array}{*{20}l} { \circledcirc } \hfill \\ \end{array} } \hfill \\ \end{array} } \hfill \\ \end{array} }}_{7(d)N}$$	[1068.685, 1069.613]	[185.6137, 185.6256]
$$T^{{\begin{array}{*{20}l} {\begin{array}{*{20}l} {\begin{array}{*{20}l} { \circledcirc } \hfill \\ \end{array} } \hfill \\ \end{array} } \hfill \\ \end{array} }}_{8(d)N}$$	[1068.681, 1069.609]	[185.6107, 185.6226]
$$T^{{\begin{array}{*{20}l} {\begin{array}{*{20}l} {\begin{array}{*{20}l} { \circledcirc } \hfill \\ \end{array} } \hfill \\ \end{array} } \hfill \\ \end{array} }}_{9(d)N}$$	[1068.682, 1069.610]	[185.6119, 185.6238]
$$T^{{\begin{array}{*{20}l} {\begin{array}{*{20}l} {\begin{array}{*{20}l} { \circledcirc } \hfill \\ \end{array} } \hfill \\ \end{array} } \hfill \\ \end{array} }}_{10(d)N}$$	[1068.684, 1069.612]	[185.6131, 185.6250]
$$T^{{\begin{array}{*{20}l} {\begin{array}{*{20}l} {\begin{array}{*{20}l} { \circledcirc } \hfill \\ \end{array} } \hfill \\ \end{array} } \hfill \\ \end{array} }}_{11(d)N}$$	[1068.681, 1069.609]	[185.6111, 185.6230]
$$T^{{\begin{array}{*{20}l} {\begin{array}{*{20}l} {\begin{array}{*{20}l} { \circledcirc } \hfill \\ \end{array} } \hfill \\ \end{array} } \hfill \\ \end{array} }}_{12(d)N}$$	[1068.682, 1069.610]	[185.6117, 185.6236]

**Table 10:** Descriptive statistics for simulation study.

Parameters	Neutrosophic values
$N$	1000
$$n_{N}$$	[20, 20]
$M_{yN}$	[196.0874, 633.9646]
$M_{xN}$	[100.8547, 325.1492]
$f_{yN} (M_{yN} )$	[0.005558005, 0.00197924]
$f_{xN} (M_{xN} )$	[0.008301677, 0.002986856]
$$\rho_{yxN}$$	[0.756, 0.764]
$C_{{M_{yN} }}$	[0.9175533, 0.7969601]
$C_{{M_{xN} }}$	[1.194368, 1.029682]
$M_{RN}$	[180.3312, 547.1558]
$Q_{DN}$	[31.52584, 86.08162]
$Q_{AN}$	[105.1535, 335.2541]
$Q_{RN}$	[63.05167, 172.1632]
$T_{MN}$	[103.0041, 330.2016]
$H_{LN}$	[104.1502, 336.6766]
$D_{MN}$	[106.1327, 336.1194]

As indicated by the indeterminacy interval findings from (23) for the whole data set, the neutrosophic generalized estimator $T_{i(d)N} ,$ is more efficient than the other suggested estimators studied. Also, the indeterminacy interval findings show that the estimator $\hat{M}_{{D_{4N} }}$ is superior to all other estimators except $T_{i(d)N}$ for the neutrosophic population, with a moderate or low correlation between the research variable and the supplementary variable (regardless of correlation is positive or negative).

### Simulation study

We evaluate the suggested estimators' efficiency using simulated neutrosophic data, such as $Y_{N}$ and $X_{N}$ are neutrosophic random variates (NRV). We generate two sets of neutrosophic random numbers of $N = 1000$, which are $x^{\prime}_{N}$ and $y^{\prime}_{N}$ from neutrosophic bivariate gamma distribution using the R programming language. Additionally, motivated by the simulated population generation strategies used by ^55^, we generate the population's $U$ transformed variables using $\rho_{yxN} = \left[ {0.7573323, \, 0.772941} \right]$, $\sigma_{yN}^{2} = \left[ {61.55041, \, 174.2082} \right]$, $\sigma_{xN}^{2} = \left[ {36.45664, \, 100.2741} \right],$

$$\mu_{yN} = \left[ {21.39525, \, 43.65861} \right],$$ $$\mu_{xN} = \left[ {12.30978, \, 24.24975} \right]$$ as $$Y_{N} = \mu_{yN} + \sigma_{yN} \left[ {\rho_{yxN} x^{\prime}_{N} + \left( {\sqrt {1 - \rho_{yxN}^{2} } } \right)y^{\prime}_{N} } \right]$$ and $$X_{N} = \mu_{xN} + \sigma_{yN} x^{\prime}_{N}$$.

where $\rho_{yxN} \in [\rho_{yxL} ,\rho_{yxU} ]$, $y^{\prime}_{N} \in [y^{\prime}_{L} ,y^{\prime}_{U} ]$, $Y_{N} \in [Y_{L} ,Y_{U} ]$, $\mu_{yN} \in [\mu_{yL} ,\mu_{yU} ]$, $\sigma_{yN}^{2} \in [\sigma_{yL}^{2} ,\sigma_{yU}^{2} ]$, and $x^{\prime}_{N} \in [x^{\prime}_{L} ,x^{\prime}_{U} ]$, $X_{N} \in [X_{L} ,X_{U} ]$,$\mu_{xN} \in [\mu_{xL} ,\mu_{xU} ]$ and $\sigma_{xN}^{2} \in [\sigma_{xL}^{2} ,\sigma_{xU}^{2} ]$.

Table 11 summarises the findings of the simulated data set utilized to evaluate the suggested estimators' efficiency to that of traditional estimators under neutrosophic statistics. Tables 12, 13, 14, 15 and 16 contain the percent relative efficiency of neutrosophic estimators. The analysis by simulated data also verifies that $T_{i(d)N}$, is the most efficient estimator. The simulation results suggest that the neutrosophic generalized estimator $T_{i(d)N}$, produces more accurate and precise findings than other estimators. All estimators are unbiased (up to the first order of approximation), efficient, and reliable.

**Table 11:** PRE’s of proposed neutrosophic estimators to $\hat{M}_{0N}$.

Estimators	PRE
$$\hat{M}_{0N}$$	[100, 100]
$\hat{M}_{RN}$	[137.6957, 143.8641]
$\hat{M}_{EN}$	[227.52, 232.4357]
$\hat{M}_{{D_{0N} }}$	[233.3918, 240.2091]
$\hat{M}_{{D_{1N} }}$	[234.4232, 240.9871]
$\hat{M}_{{D_{2N} }}$	[234.4415, 240.9974]
$\hat{M}_{{D_{3N} }}$	[235.7074, 241.9697]
$\hat{M}_{{D_{4N} }}$	[242.8629, 247.422]

**Table 12:** PREs of proposed neutrosophic estimators $T^{ \oplus }_{i(d)N}$ to $\hat{M}_{0N}$.

Estimators	PRE
$$T^{{\begin{array}{*{20}l} { \ominus } \hfill \\ \end{array} }}_{1(d)N}$$	[255.0274, 257.0464]
$$T^{{\begin{array}{*{20}l} { \ominus } \hfill \\ \end{array} }}_{2(d)N}$$	[255.348, 257.1181]
$$T^{{\begin{array}{*{20}l} { \ominus } \hfill \\ \end{array} }}_{3(d)N}$$	[255.0201, 257.0472]
$$T^{{\begin{array}{*{20}l} { \ominus } \hfill \\ \end{array} }}_{4(d)N}$$	[255.0029, 257.0399]
$$T^{{\begin{array}{*{20}l} { \ominus } \hfill \\ \end{array} }}_{5(d)N}$$	[255.0189, 257.0428]
$$T^{{\begin{array}{*{20}l} { \ominus } \hfill \\ \end{array} }}_{6(d)N}$$	[255.3209, 257.1355]
$$T^{{\begin{array}{*{20}l} { \ominus } \hfill \\ \end{array} }}_{7(d)N}$$	[255.343, 257.1159]
$$T^{{\begin{array}{*{20}l} { \ominus } \hfill \\ \end{array} }}_{8(d)N}$$	[254.9967, 257.0439]
$$T^{{\begin{array}{*{20}l} { \ominus } \hfill \\ \end{array} }}_{9(d)N}$$	[255.0344, 257.0469]
$$T^{{\begin{array}{*{20}l} { \ominus } \hfill \\ \end{array} }}_{10(d)N}$$	[255.3343, 257.1161]
$$T^{{\begin{array}{*{20}l} { \ominus } \hfill \\ \end{array} }}_{11(d)N}$$	[255.0043, 257.0431]
$$T^{{\begin{array}{*{20}l} { \ominus } \hfill \\ \end{array} }}_{12(d)N}$$	[255.0187, 257.0441]

**Table 13:** PRE’s of proposed neutrosophic estimators $T^{ \oplus }_{i(d)N}$ to $\hat{M}_{0N}$.

Estimators	PRE
$T^{ \oplus }_{1(d)N}$	[253.457, 255.6164]
$T^{ \oplus }_{2(d)N}$	[253.6734, 255.6645]
$T^{ \oplus }_{3(d)N}$	[253.452, 255.617]
$T^{ \oplus }_{4(d)N}$	[253.4404, 255.6121]
$T^{ \oplus }_{5(d)N}$	[253.4513, 255.614]
$T^{ \oplus }_{6(d)N}$	[253.6551, 255.6761]
$T^{ \oplus }_{7(d)N}$	[253.67, 255.663]
$T^{ \oplus }_{8(d)N}$	[253.4363, 255.6148]
$T^{ \oplus }_{9(d)N}$	[253.4617, 255.6168]
$T^{ \oplus }_{10(d)N}$	[253.6641, 255.6631]
$T^{ \oplus }_{11(d)N}$	[253.4414, 255.6142]
$T^{ \oplus }_{12(d)N}$	[253.4511, 255.6149]

**Table 14:** PRE’s of proposed neutrosophic estimators $T^{ \oplus }_{i(d)N}$ to $\hat{M}_{0N}$.

Estimators	PRE
$T^{ \otimes }_{1(d)N}$	[276.5886, 272.7765]
$T^{ \otimes }_{2(d)N}$	[277.1863, 272.9037]
$T^{ \otimes }_{3(d)N}$	[276.5749, 272.7779]
$T^{ \otimes }_{4(d)N}$	[276.5429, 272.765]
$T^{ \otimes }_{5(d)N}$	[276.5728, 272.7701]
$T^{ \otimes }_{6(d)N}$	[277.1358, 272.9345]
$T^{ \otimes }_{7(d)N}$	[277.1769, 272.8997]
$T^{ \otimes }_{8(d)N}$	[276.5314, 272.772]
$T^{ \otimes }_{9(d)N}$	[276.6017, 272.7773]
$T^{ \otimes }_{10(d)N}$	[277.1607, 272.9]
$T^{ \otimes }_{11(d)N}$	[276.5455, 272.7706]
$T^{ \otimes }_{12(d)N}$	[276.5723, 272.7723]

**Table 15:** PRE’s of proposed neutrosophic estimators $T^{ \oplus }_{i(d)N}$ to $\hat{M}_{0N}$.

Estimators	PRE
$$T^{{\begin{array}{*{20}l} {\begin{array}{*{20}l} { \circledast } \hfill \\ \end{array} } \hfill \\ \end{array} }}_{1(d)N}$$	[297.4101, 288.5158]
$$T^{{\begin{array}{*{20}l} {\begin{array}{*{20}l} { \circledast } \hfill \\ \end{array} } \hfill \\ \end{array} }}_{2(d)N}$$	[298.6929, 288.7828]
$$T^{{\begin{array}{*{20}l} {\begin{array}{*{20}l} { \circledast } \hfill \\ \end{array} } \hfill \\ \end{array} }}_{3(d)N}$$	[297.3808, 288.5187]
$$T^{{\begin{array}{*{20}l} {\begin{array}{*{20}l} { \circledast } \hfill \\ \end{array} } \hfill \\ \end{array} }}_{4(d)N}$$	[297.3124, 288.4917]
$$T^{{\begin{array}{*{20}l} {\begin{array}{*{20}l} { \circledast } \hfill \\ \end{array} } \hfill \\ \end{array} }}_{5(d)N}$$	[297.3763, 288.5024]
$$T^{{\begin{array}{*{20}l} {\begin{array}{*{20}l} { \circledast } \hfill \\ \end{array} } \hfill \\ \end{array} }}_{6(d)N}$$	[298.5841, 288.8476]
$$T^{{\begin{array}{*{20}l} {\begin{array}{*{20}l} { \circledast } \hfill \\ \end{array} } \hfill \\ \end{array} }}_{7(d)N}$$	[298.6726, 288.7745]
$$T^{{\begin{array}{*{20}l} {\begin{array}{*{20}l} { \circledast } \hfill \\ \end{array} } \hfill \\ \end{array} }}_{8(d)N}$$	[297.288, 288.5065]
$$T^{{\begin{array}{*{20}l} {\begin{array}{*{20}l} { \circledast } \hfill \\ \end{array} } \hfill \\ \end{array} }}_{9(d)N}$$	[297.4381, 288.5176]
$$T^{{\begin{array}{*{20}l} {\begin{array}{*{20}l} { \circledast } \hfill \\ \end{array} } \hfill \\ \end{array} }}_{10(d)N}$$	[298.6376, 288.7752]
$$T^{{\begin{array}{*{20}l} {\begin{array}{*{20}l} { \circledast } \hfill \\ \end{array} } \hfill \\ \end{array} }}_{11(d)N}$$	[297.318, 288.5035]
$$T^{{\begin{array}{*{20}l} {\begin{array}{*{20}l} { \circledast } \hfill \\ \end{array} } \hfill \\ \end{array} }}_{12(d)N}$$	[297.3753, 288.5071]

**Table 16:** PRE’s of proposed neutrosophic estimators $T^{ \oplus }_{i(d)N}$ to $\hat{M}_{0N}$.

Estimators	PRE
$$T^{{\begin{array}{*{20}l} {\begin{array}{*{20}l} {\begin{array}{*{20}l} { \circledcirc } \hfill \\ \end{array} } \hfill \\ \end{array} } \hfill \\ \end{array} }}_{1(d)N}$$	[302.4588, 291.9083]
$$T^{{\begin{array}{*{20}l} {\begin{array}{*{20}l} {\begin{array}{*{20}l} { \circledcirc } \hfill \\ \end{array} } \hfill \\ \end{array} } \hfill \\ \end{array} }}_{2(d)N}$$	[303.4742, 292.1171]
$$T^{{\begin{array}{*{20}l} {\begin{array}{*{20}l} {\begin{array}{*{20}l} { \circledcirc } \hfill \\ \end{array} } \hfill \\ \end{array} } \hfill \\ \end{array} }}_{3(d)N}$$	[302.4356, 291.9106]
$$T^{{\begin{array}{*{20}l} {\begin{array}{*{20}l} {\begin{array}{*{20}l} { \circledcirc } \hfill \\ \end{array} } \hfill \\ \end{array} } \hfill \\ \end{array} }}_{4(d)N}$$	[302.3813, 291.8894]
$$T^{{\begin{array}{*{20}l} {\begin{array}{*{20}l} {\begin{array}{*{20}l} { \circledcirc } \hfill \\ \end{array} } \hfill \\ \end{array} } \hfill \\ \end{array} }}_{5(d)N}$$	[302.432, 291.8977]
$$T^{{\begin{array}{*{20}l} {\begin{array}{*{20}l} {\begin{array}{*{20}l} { \circledcirc } \hfill \\ \end{array} } \hfill \\ \end{array} } \hfill \\ \end{array} }}_{6(d)N}$$	[303.3882, 292.1677]
$$T^{{\begin{array}{*{20}l} {\begin{array}{*{20}l} {\begin{array}{*{20}l} { \circledcirc } \hfill \\ \end{array} } \hfill \\ \end{array} } \hfill \\ \end{array} }}_{7(d)N}$$	[303.4582, 292.1106]
$$T^{{\begin{array}{*{20}l} {\begin{array}{*{20}l} {\begin{array}{*{20}l} { \circledcirc } \hfill \\ \end{array} } \hfill \\ \end{array} } \hfill \\ \end{array} }}_{8(d)N}$$	[302.3619, 291.901]
$$T^{{\begin{array}{*{20}l} {\begin{array}{*{20}l} {\begin{array}{*{20}l} { \circledcirc } \hfill \\ \end{array} } \hfill \\ \end{array} } \hfill \\ \end{array} }}_{9(d)N}$$	[302.481, 291.9097]
$$T^{{\begin{array}{*{20}l} {\begin{array}{*{20}l} {\begin{array}{*{20}l} { \circledcirc } \hfill \\ \end{array} } \hfill \\ \end{array} } \hfill \\ \end{array} }}_{10(d)N}$$	[303.4305, 292.1112]
$$T^{{\begin{array}{*{20}l} {\begin{array}{*{20}l} {\begin{array}{*{20}l} { \circledcirc } \hfill \\ \end{array} } \hfill \\ \end{array} } \hfill \\ \end{array} }}_{11(d)N}$$	[302.3857, 291.8987]
$$T^{{\begin{array}{*{20}l} {\begin{array}{*{20}l} {\begin{array}{*{20}l} { \circledcirc } \hfill \\ \end{array} } \hfill \\ \end{array} } \hfill \\ \end{array} }}_{12(d)N}$$	[302.4313, 291.9015]

**Figure 4:**
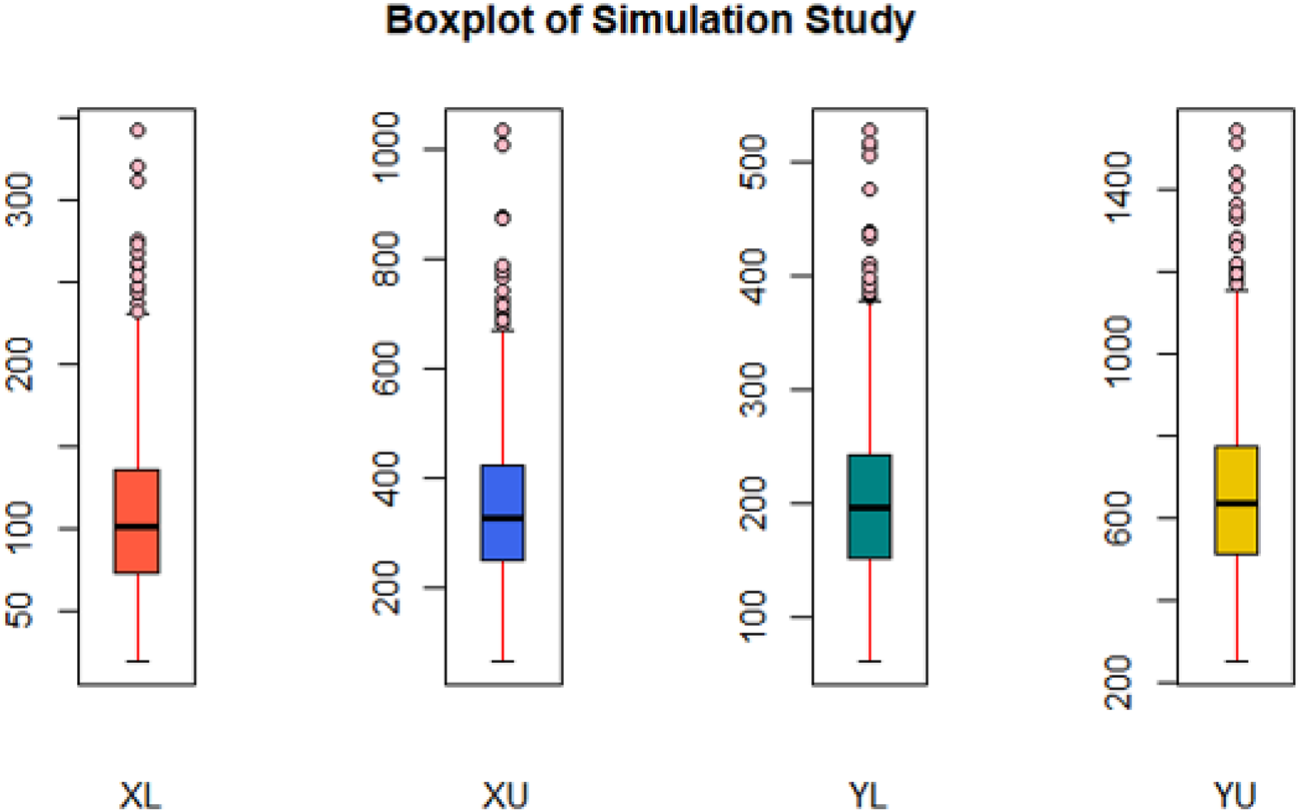
Boxplot of simulated data for each variable.

**Figure 5:**
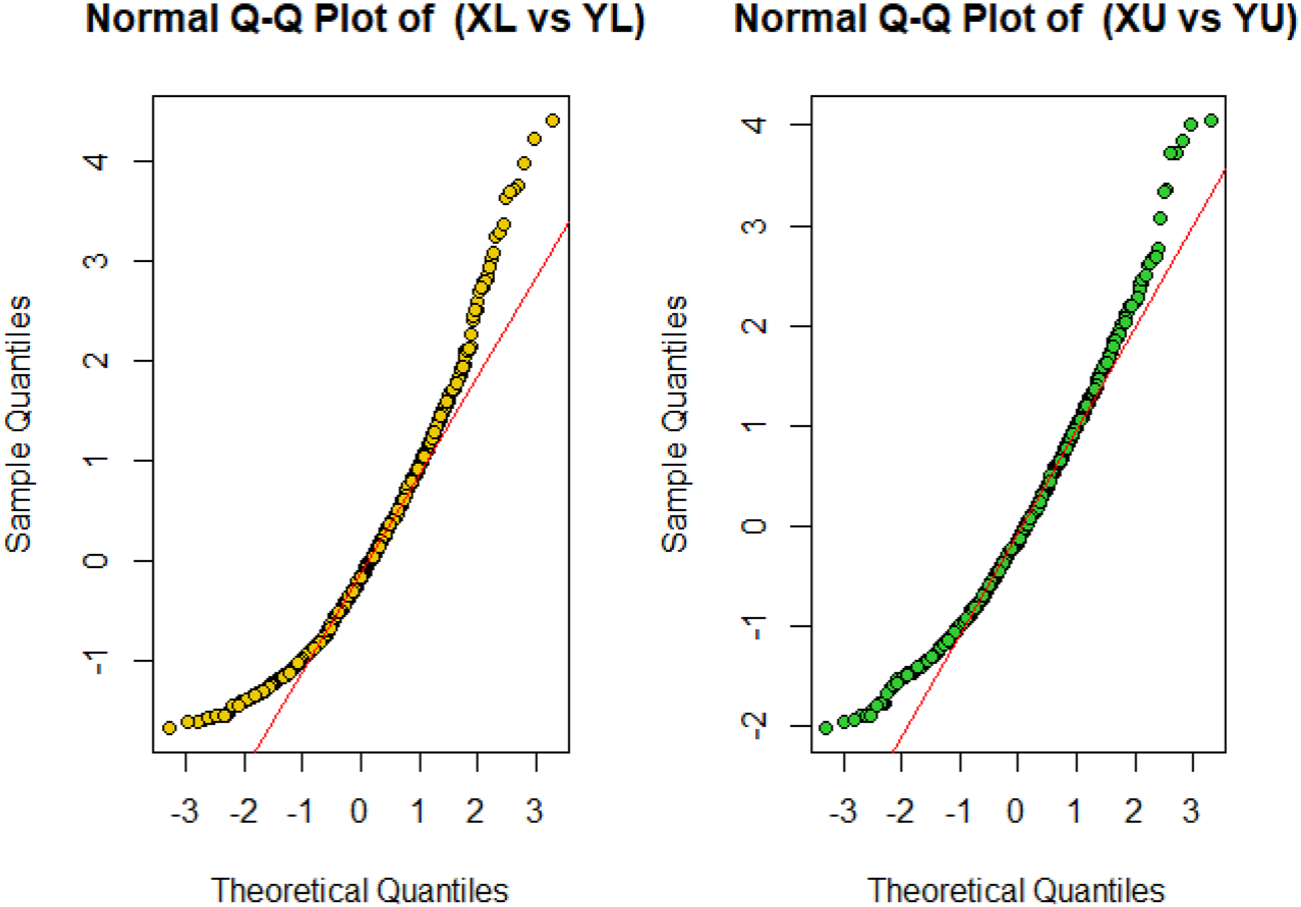
Normal Q-Q plot of simulated data for variable $X_{N}$ and $Y_{N}$.

In Figs. 4 and 5, we have displayed the performance of simulated data by using boxplot and Q-Q plot, respectively. The boxplots show that data is positively skewed, which implies that the median is closer to the lower or bottom quartile. The boxplot with points beyond the whiskers indicates that the data has a few outliers. Normal Q-Q plot displays that the top end of the Q-Q plot deviates from the straight line, while the lower end follows the straight line, and that the curves have a more prominent tail to the right, indicating that they are right-skewed (or positively skewed).

## Conclusion

Our study introduced neutrosophic estimators for accurately estimating the population median in datasets with uncertain, unclear values. Through precise additional variable measurements under simple random sampling, we developed improved neutrosophic estimators, evaluated them for bias and MSE, and demonstrated their superiority. Our proposed estimators offer the advantage of modelling uncertainty and vagueness inherent in many real-world scenarios, allowing for more flexible and nuanced decision-making processes. However, their disadvantage lies in the complexity of mathematical models and computational processes required, which can lead to increased computational costs and challenges in interpretation and precise quantification. We recommend these advanced estimators for future applications and highlight the ongoing need for research to enhance estimator effectiveness for various neutrosophic data types and sampling methods. Furthermore, future work will extend to multiple sampling designs, such as systematic, successive, and double sampling.

## Data Availability

Processed data are available from the corresponding author upon reasonable request.
